# A Novel R2R3-MYB Transcription Factor FtMYB22 Negatively Regulates Salt and Drought Stress through ABA-Dependent Pathway

**DOI:** 10.3390/ijms232314549

**Published:** 2022-11-22

**Authors:** Haixia Zhao, Panfeng Yao, Jiali Zhao, Huala Wu, Shuang Wang, Ying Chen, Mufan Hu, Tao Wang, Chenglei Li, Qi Wu

**Affiliations:** 1 College of Life Science, Sichuan Agricultural University, Ya’an 625014, China; 2 State Key Laboratory of Aridland Crop Science, Gansu Agricultural University, Lanzhou 730070, China

**Keywords:** Tartary buckwheat, FtMYB22, transcription factor, abiotic stress, ABA

## Abstract

Tartary buckwheat (*Fagopyrum tataricum* Gaertn.) is a coarse cereal with strongly abiotic resistance. The MYB family plays a regulatory role in plant growth, development, and responses to biotic and abiotic stresses. However, the characteristics and regulatory mechanisms of MYB transcription factors in Tartary buckwheat remain unclarified. Here, this study cloned the *FtMYB22* gene from Tartary buckwheat, and investigated its involvement in responding to individual water deficit and salt stress in *Arabidopsis*. Sequence analysis highlighted that the N-termini of FtMYB22 contained two highly conserved SANT domains and one conserved domain from the SG20 subfamily. Nucleus-localized FtMYB22 did not have individual transcriptional activation activity. Water deficiency and salt stress induced the high expression of the *GUS* gene, which was driven by the promoter of *FtMYB22*. Yeast stress experiments showed that the overexpression of *FtMYB22* significantly reduced the growth activity of transgenic yeast under water deficit or salt stress. Consistently, the overexpression of *FtMYB22* reduced the salt and water deficit stress resistance of the transgenic plants. In addition, physiological parameters showed that transgenic plants had lower proline and antioxidant enzyme activity under stress conditions. Compared to the wild-type (WT), transgenic plants accumulated more malondialdehyde (MDA), H_2_O_2_, and O_2_^−^; they also showed higher ion permeability and water loss rates of detached leaves under stress treatments. Notably, FtMYB22 was involved in plant stress resistance through an ABA-dependent pathway. Under stress conditions, the expression of *RD29A*, *RD29B*, *PP2CA*, *KIN1*, *COR15A*, and other genes in response to plant stress in transgenic lines was significantly lower than that in the WT (*p* < 0.05). Furthermore, yeast two-hybrid assay showed that there was a significant interaction between FtMYB22 and the ABA receptor protein RCAR1/2, which functioned in the ABA signal pathway. Altogether, FtMYB22, as a negative regulator, inhibited a variety of physiological and biochemical reactions, affected gene expression and stomatal closure in transgenic plants through the ABA-dependent pathway, and reduced the tolerance of transgenic *Arabidopsis* to water deficiency and salt stress. Based on these fundamental verifications, further studies would shed light on the hormone signal response mechanism of FtMYB22.

## 1. Introduction

Abiotic stresses, such as drought, high salinity, and extreme temperature, severely affect growth, development, and crop yield [1]. These stresses have caused around more than 50% of yield losses of major crops across the whole world [2]. Therefore, it is essential to further understand how plants respond to these environmental factors. Over the millennia, plants have evolved complex regulatory mechanisms at the physiological, metabolic, morphological, and molecular levels to promote stress tolerance [3,4]. At the molecular level, stress-induced regulatory proteins, including the transcription factor (TF) MYB, bHLH, NAC, DREB, and WRKY families, are universally activated to mediate plant tolerance to adverse stress [5,6]. In addition to generate functional proteins that directly protect cells from stress-induced damage, TFs also can act as signaling molecules to regulate the transcription levels of downstream target genes [7,8].

MYB, a large and extensively studied family of TFs in plants, plays a vital role in plant growth, development, metabolism, and stress response [9]. According to the number of adjacent repeats in their DNA-binding domains, plant MYB TFs can be divided into four different subfamilies, which are termed 1R-MYB, R2R3-MYB, 3R-MYB, and 4R-MYB. The R2R3-MYB protein forms the largest MYB subfamily and is the most widely studied [9,10]. Numerous studies have demonstrated that the overproduction of stress-related R2R3-MYB TFs can alter the abiotic stress tolerance of transgenic plants in many species. For example, the ectopic expression of *AtMYB44* conferred abiotic stress tolerance to transgenic *Arabidopsis* by enhancing stomatal closure [11]. Studies have shown that AtMYB2, which belongs to the SG20 subfamily, acts as a transcriptional activator in the ABA signal transduction pathway under drought stress conditions [12]. *OsMYB48-1* plays a positive role in drought and salinity tolerance by regulating stress-induced ABA synthesis [13]. *TaMYBsm1* genes increased the resistance of transgenic *Arabidopsis* to drought stress by upregulating *DREB2A*, *P5CS1*, and *RD29A* [14]. In contrast, there are also some TFs that play a negative regulatory role in plant stress tolerance. The overexpression of *GmMYB3a* negatively regulates salt-alkali tolerance in soybean [15]. Loss of the AtMYB73 caused hyper-induction of the *SOS1* and *SOS3* genes, and *AtMYB73* mutants showed higher survival rates than WTs under high salinity conditions [16].

Tartary buckwheat is a dicotyledonous plant of the Fagopyrum genus in the Polygonaceae. It is also a medicinal and edible homology resource, belonging to minor coarse cereals originating from southwestern China. Tartary buckwheat mainly contains rutin, quercetin, kaempferol and other flavonols, as well as anthocyanins, proanthocyanidins, and their derivatives [17]. It not only has high nutritive and healthcare value, but also is well-known for its excellent stress resistance. Recently, the functions of stress-resistant TFs, such as MYB, bZIP, NAC, bHLH and WRKYs, have been well studied using the abundant transcriptome sequencing data available for Tartary buckwheat. However, there are still a large number of stress-associated TF genes, especially in the MYB TF family, that require in-depth excavation and systematic identification. Our previous studies have shown that *FtMYB22* (accession number: KT285536.1), an SG20 subfamily of the MYB family gene, may play a central role in responding to salt and water deficiency stress during plant growth and development [18]. To further validate this speculation, we cloned the *FtMYB22* gene and investigated its role in water deficiency and salinity tolerance. We also discussed its potential regulation mechanism in this study.

## 2. Results

### 2.1. FtMYB22 Is a Potential Regulator for the Flavonol Biosynthetic Pathway in Tartary Buckwheat

**Figure 1:**
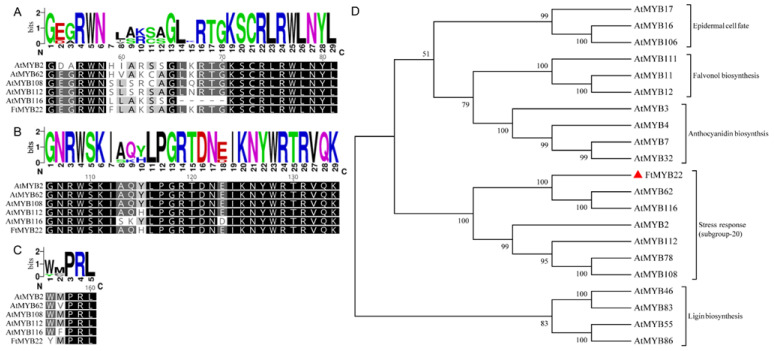
Multiple sequence alignments for conserved domains and the phylogenetic tree of FtMYB22. (**A**) R2-conserved domain. (**B**) R3-conserved domain. (**C**) WxPRL, SG20 subfamily-conserved domain. (**D**) The phylogenetic tree generated from the alignment of LFtMYB22 and *Arabidopsis* MYBs using the neighbor-joining (NJ) method. FtMYB22 is highlighted with a red triangle. AtMYB17: At3g61250, AtMYB16: At5g15310, AtMYB106: At3g01140, AtMYB46: At5g12870, AtMYB83: At3g08500, AtMYB55: At4g01680, AtMYB86: At5g26660, AtMYB3: At1g22640, AtMYB4: At4g38620, AtMYB7: At2g16720, AtMYB32: At4g34990, AtMYB111: At5g49330, AtMYB11: At3g62610, AtMYB12: At2g47460, AtMYB62: At1g68320, AtMYB116: At1g25340, AtMYB2: At2g47190, AtMYB112: At1g48000, AtMYB78: At5g49620, AtMYB108: At3g06490. Identical and similar amino acid residues are marked in black and grey, respectively.

A previous study had isolated eight *MYB* genes that might have been involved in the stress response based on the Tartary buckwheat flower transcriptome data [18]. Among them, the *FtMYB22* gene encodes a putative protein of 267 amino acids with a calculated molecular mass of 30.93 kD and a pI of 5.69. According to the NCBI BLAST results, the deduced amino acid sequence of FtMYB22 shares 49% identity with VvMYB108 and 56% identity with CqMYB62. The primary structure analysis showed that FtMYB22 contains two incomplete MYB repeats that approximately match with the R2 and R3 repeats in the N-terminal (Figure 1A,B), which function in sequence-specific DNA binding. Extremely conserved W residues, which are possibly involved in folding the DNA-binding domain, are also present in FtMYB22 [19]. In addition, WxPRL, a typical conserved motif of R2R3-MYB subgroup-20, was found in the downstream of the R3 repeat (Figure 1C) [20]. Phylogenetic analysis indicated that FtMYB22 and stress-associated R2R3-MYB TFs were categorized into the same clusters belonging to *Arabidopsis* subgroup-20 (Figure 1D), which revealed the possibility of functional conservation among them.

### 2.2. FtMYB22 Localizes to the Nucleus and Acts as a Transcriptional Inhibitor

**Figure 2:**
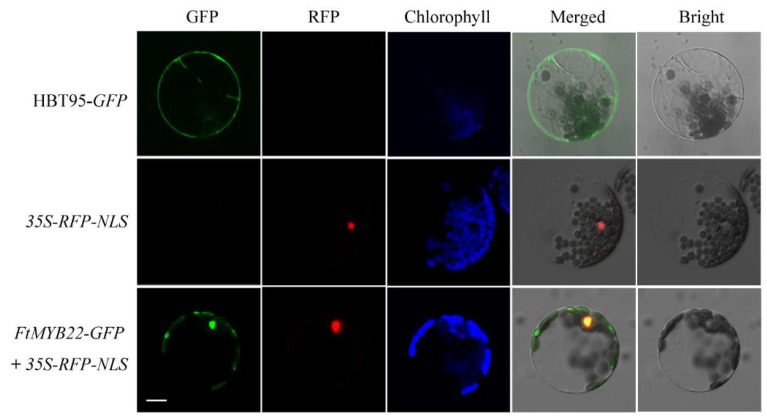
Subcellular localization of FtMYB22. *Arabidopsis* protoplasts transformed with *35S-RFP-NLS* and *HBT95-GFP* were used as the positive control and negative control, respectively. Co-transformed *Arabidopsis* protoplasts with *FtMYB22-GFP* and *35S-RFP-NLS* were used for subcellular localization. The chloroplasts autofluorescence (chlorophyll) were also visualized by a laser scanning confocal microscopy. Scale bar: 10 µm.

FtMYB22 was predicted to localize in the nucleus (95.7%) and cytoplasm (4.3%), according to GenScript^®^ software (https://www.genscript.com/psort.html, accessed on 12 August 2020). Subsequently, the subcellular localization assay was carried out in *Arabidopsis* protoplast cells. Results showed that the FtMYB22-GFP fluorescence colocalized with RFP (red fluorescent proteins) fusing with a nuclear localization signal (RFP-NLS) (Figure 2). The green fluorescent signal was also detected in the cytoplasm. These results were consistent with the prediction and confirmed that FtMYB22 protein mainly localized in the nucleus and partially in the cytoplasm. Additionally, the transcriptional activation activity assay was performed to determine the corresponding transcriptional activation activity of FtMYB22. Upon shift cultivation under the normal growth condition (SD/-Trp medium, Figure S1A–D) to the inducible condition (SD/−Trp/−His medium), it was shown that only transformed yeast with pBridge-GmMYBJ6 (positive control) grew normally on SD/−His/−Trp medium (Figure S1E,F), which suggested that FtMYB22 does not have individual transcriptional activation activity in vitro.

### 2.3. Response of FtMYB22 Promoter to Stress Conditions

To further investigate the response of *FtMYB22* to environmental factors, the promoter of the *FtMYB22* gene was cloned and named *ProFtMYB22*. Sequence analysis indicated that there were three transcription start sites with a probability of more than 80% at the 5′ termini of the *FtMYB22*, which were located at −87 bp, −297 bp, and −846 bp, respectively. Additionally, a variety of *cis*-acting elements were predicted in the *ProFtMYB22* sequence, and mainly included elements involved in light response, such as GAG-motif, LAMP-element, TCCC-motif, and H-box; elements responding to stress response, such as TC-rich repeats and MBS (MYB binding site); and elements for hormone response, such as CGTCA-motif, G-box, and ABRE (ABA-responsive elements) (Table S2).

**Figure 3:**
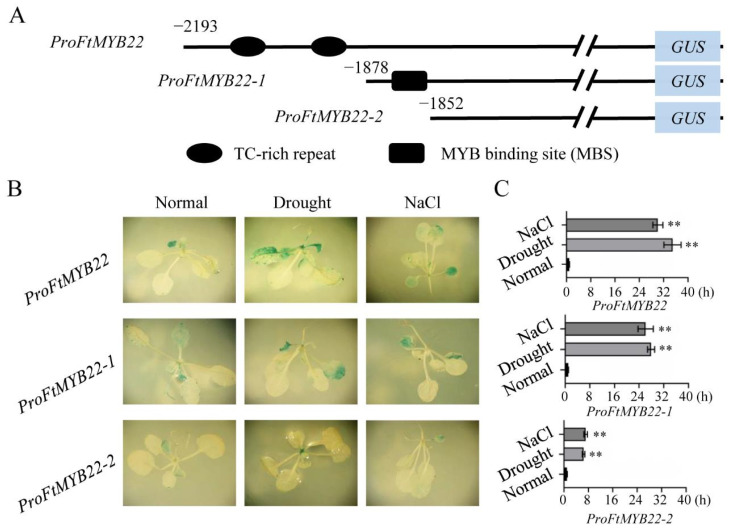
Response analyses of *ProFtMYB22* to water deficit and salt stress. (**A**) Truncated model of *ProFtMYB22*. (**B**) GUS staining in *ProFtMYB22* transgenic plants under abiotic stress as indicated. (**C**) Expression levels of *GUS* in *ProFtMYB22* transgenic plants following indicated treatments. Normal: the normal condition. Data are presented as mean and SD values of three independent experiments. Error bars represent standard errors. ** means extremely significant difference at *p* < 0.01 level.

Subsequently, the *ProFtMYB22* sequence was truncated to investigate the stress response of these elements according to the element prediction (TC-rich repeats and MBS), and were termed *ProFtMYB22, ProFtMYB22-1*, and *ProFtMYB22-2* (Figure 3A). T3 transgenic lines with *ProFtMYB22, ProFtMYB22-1*, and *ProFtMYB22-2* were selected for further analysis (Figure S2). Figure 3B showed that the truncated *ProFtMYB22* did not affect its activity in transgenic plants under the normal condition. Under NaCl and water deficit conditions, *ProFtMYB22* and *ProFtMYB22-1* showed similar responses with the increase in *GUS* expression levels to 30.1-fold (*ProFtMYB22* under NaCl), 34.9-fold (*ProFtMYB22* under water deficit), 26.1-fold (*ProFtMYB22-1* under NaCl), and 27.9-fold (*ProFtMYB22-1* under water deficit), respectively, when compared to those under the normal condition (control). These results highlighted that *ProFtMYB22* could respond to stress, and its TC-rich repeats had no significant effect. In contrast, following the treatments of NaCl and water deficit, the expression levels of the *GUS* gene in *ProFtMYB22-2* transgenic plants increased by 7.1- and 6.4-fold, respectively, when compared to the normal condition(Figure 3C). These results illustrated that the MBS element was the main domain of the *ProFtMYB22* in response to environmental stress.

### 2.4. Overexpression of FtMYB22 Reduces the Tolerance of Transgenic Yeast to Multiple Stresses

**Figure 4:**
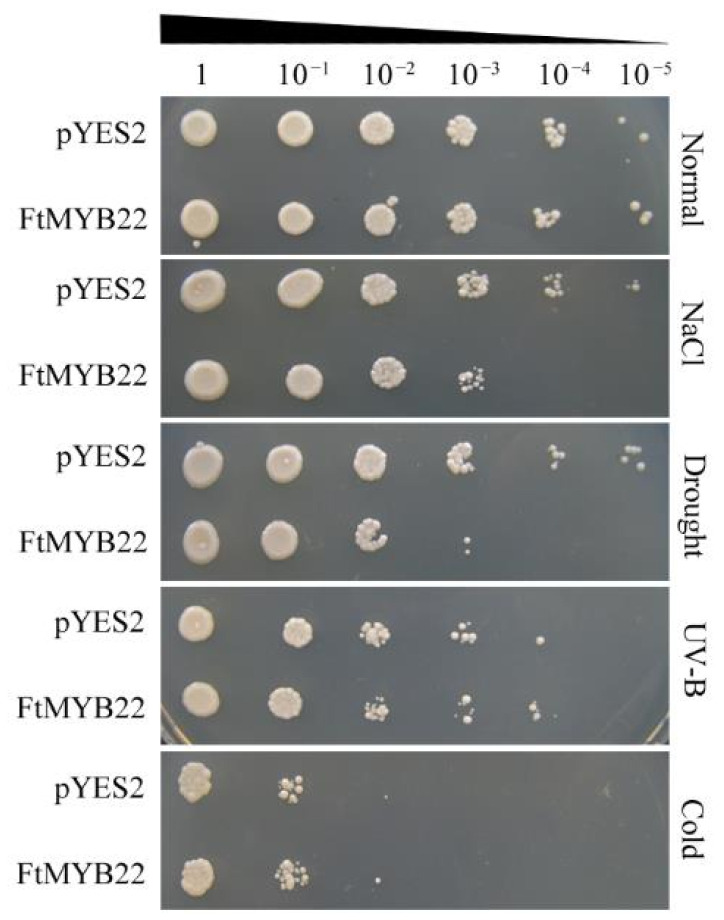
Growth phenotype of *FtMYB22* transgenic yeast under stress conditions as indicated. The yeast cells containing recombinant plasmids were serially diluted by 10, 100, 1000, 10,000, and 100,000 folds, and then respectively cultured on SC/−Ura medium. 30% (*w*/*v*) PEG6000 (for the water deficit) or 5 M NaCl (for the salt stress) was added to the cell fluid and cultured at 30 °C for 36 h. For the UV-B stress, the yeast cells were placed in 10 W UV-B (308 nm) LED for 36 h; the illumination distance from lamp to bacterial liquid was 30 cm, and the radiation intensity was 0.1 mW/cm^2^. Yeast cells were resuspended in SC-ura medium and cultured at −20 °C for 24 h for the cold stress. For the normal control, the same amount of yeast cells was resuspended in 200 μL of sterilized water and cultured at 30 °C for 36 h.

Two transformed yeast lines with *FtMYB22* and an empty *pYES2* vector (control) were constructed. qRT-PCR (quantitative real-time PCR) analysis confirmed that the transformed *FtMYB22* was expressed in yeast (Figure S3). Subsequently, the transformed lines and control lines were evaluated under different abiotic stresses. The results indicated that there was no difference in the growth status of transgenic *FtMYB22* or *pYES2* yeast cells under the normal condition (Figure 4). Under salt or water deficit treatment, the yeast cells overexpressing *FtMYB22* could not grow properly when the cells were diluted by 10^4^ and 10^5^ times. In addition, although cold and UV-B treatments affected the growth status of the *FtMYB22*- and *pYES2*-transformed yeast lines, significant differences were not observed between them. These results showed that *FtMYB22* reduced the tolerance of transgenic yeast cells to water deficiency and salt stress.

### 2.5. Overexpression of FtMYB22 Reduces the Tolerance of Transgenic Arabidopsis to Osmotic Stress

**Figure 5:**
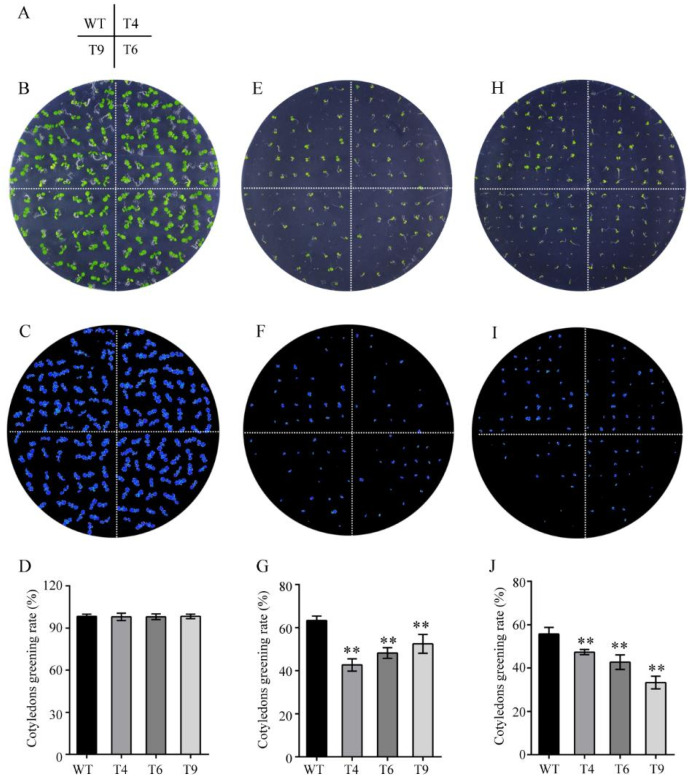
Cotyledon greening rate of FtMYB22 transgenic *Arabidopsis* (T4, T6, and T9) lines and WT lines. (**A**) The distribution of each line on the plate. (**B**–**D**) Cotyledon greening under the normal condition. (**E**–**G**) Cotyledon greening under mannitol condition. (**H**–**J**) Cotyledon greening under salt condition. (**B**,**E**,**H**) are characterized in daylight. (**C**,**F**,**I**) are characterized under chlorophyll fluorescence. WT, wild-type. Data are presented as mean ± SD values of three independent experiments. Error bars represent SD. ** means extremely significant difference at *p* < 0.01 level.

Since the expression levels of *FtMYB22* in Tartary buckwheat and yeasts can be significantly induced by salt and water deficit stress [18], transgenic *Arabidopsis* plants overexpressing *FtMYB22* were also generated to further investigate the function of *FtMYB22* in plants. Eight T0 transgenic lines were verified by PCR (Figure S4) and three homozygous T3 transgenic lines (T4, T6, and T9) with high *FtMYB22* expression levels were selected for further analysis. The effects of water deficit and salt treatment on the cotyledon greening and root growth of the transgenic plants were determined. 200 mM of mannitol and 150 mM of NaCl were each added to 1/2 MS medium to simulate water deficiency and salt stress. Under the normal condition, there was no significant difference in the cotyledon greening rate between transgenic plants and WTs during the growth period (Figure 5B–D). However, under the stress conditions, the cotyledon greening rate of both the *FtMYB22* transgenic lines and the WTs was suppressed, while greater repression in the cotyledon greening rate occurred in transgenic lines. Under the water deficit condition, WTs started to germinate 2 days after sowing, whereas transgenic lines began after 3 days. After 5 days, WTs almost reached a maximum cotyledon greening rate of 90.5%, while this rate was 54.4% for transgenic plants (Figure 5E–G). In addition, the salt treatment also showed an obvious effect on the germination of transgenic plants, and the cotyledon greening rate of the transgenic lines was consistently lower than that of the WTs after the seed germination (Figure 5H–J).

**Figure 6:**
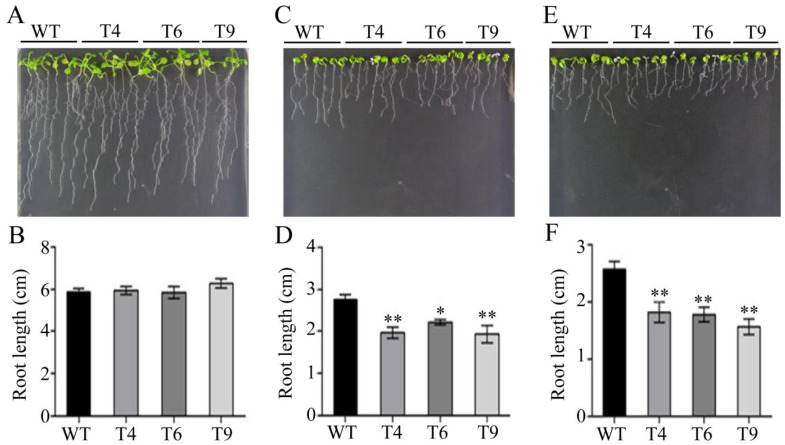
Root length of *FtMYB22* transgenic *Arabidopsis* (T4, T6, and T9) lines and WT lines. (**A**,**B**) Root length under normal condition. (**C**,**D**) Root length under mannitol condition after 7 days. (**E**,**F**) Root length under salt condition after 7 days. WT, wild-type. Data are presented as mean ± SD and calculated from three independent experiments. Error bars represent standard errors. ** means extremely significant difference at *p* < 0.01 level, * means significant difference at *p* < 0.05 level.

Similarly, there were no differences in root lengths between the transgenic lines and WTs under the normal condition (Figure 6A,B). Transgenic seedlings had shorter root lengths than WTs under the water deficit and salt conditions. The root growth of all lines was inhibited under mannitol stress. The root length under the water deficit condition was only 43.3% of that of the normal condition in the WTs. The root lengths of the three transgenic lines were only 76.1%, 83.8%, and 71.3% of those in the control group for T4, T6, and T9, respectively, which were significantly lower than those of WTs (Figure 6C,D). In addition, similar growth trends were also obtained under NaCl treatment. Under salt stress, the root growth of all lines was only 30.2% of that of the normal growth condition. These data showed that the root lengths of three transgenic lines were only 80.1%, 78.2%, and 68.8% of those in the control group under the same conditions (Figure 6E,F) for T4, T6, and T9, respectively. This further indicated that FtMYB22 played a negative regulatory role in stress response.

### 2.6. Overproduction of FtMYB22 Reduces the Tolerance of Transgenic Arabidopsis to Salt and Water Deficit Stresses

**Figure 7:**
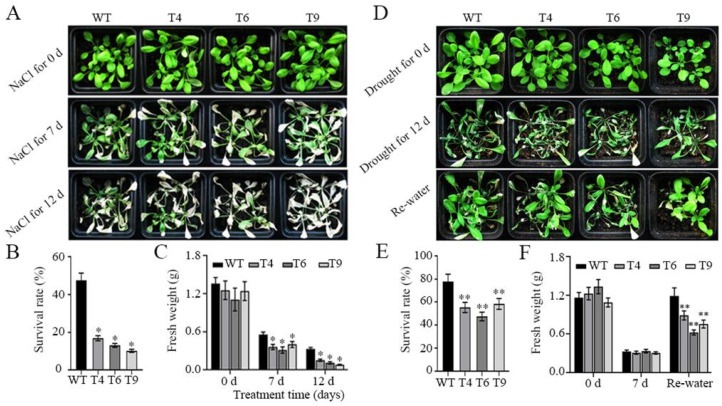
Stress tolerance of *FtMYB22* transgenic *Arabidopsis* (T4, T6, and T9) lines and WT lines under salt and drought conditions. (**A**) Phenotype of *FtMYB22* transgenic *Arabidopsis* lines under salt stress over time. (**B**) Survival rate of *FtMYB22* transgenic *Arabidopsis* lines following the salt treatment for 12 d. (**C**) Fresh weight of *FtMYB22* transgenic *Arabidopsis* lines under salt stress. (**D**) Phenotype of *FtMYB22* transgenic *Arabidopsis* lines under water deficit stress for 12 d. (**E**) Survival rate of *FtMYB22* transgenic *Arabidopsis* lines under water deficit stress for 12 d. (**F**) Fresh weight of *FtMYB22* transgenic *Arabidopsis* lines under water deficit stress for 12 d. WT, wild-type. Data are calculated from triple experiments and presented as mean ± SD. Error bars indicate standard errors. * means extremely significant difference at *p* < 0.01 level, ** means extremely significant difference at *p* < 0.01 level.

For further experiments, water deficit and salinity treatments were applied to three-week-old plants to test the growth performance of transgenic and WT plants grown in the soil. For the salt treatment assay, the transgenic plants overexpressing *FtMYB22* did not show any differences compared to WTs under the normal irrigation condition (Figure 7A). After treatment for 7 days, the leaves of all plants were seriously curled and shrunken, including WTs and transgenic plants. The transgenic plants were more seriously withered and whitened than the WTs. 12 days later, this difference between transgenics and WTs was more pronounced, and only approximately 20% of transgenic plants survived. By comparison, the survival rate of the WTs was over 45%, and some of them still grew with green leaves (Figure 7B). Furthermore, the fresh weight of transgenic plants was lower than WTs at different stages of treatment (Figure 7C). Similarly, for the water deficit assay, WTs exhibited a stronger growth recovery than transgenic plants after re-watering for 5 days, while only approximately 50% of the transgenic plants survived (Figure 7D). In contrast, more than 75% of the WTs still stayed alive (Figure 7E). Unlike the salt treatment, the fresh weights of the transgenic plants were almost the same as the WTs following water deficit treatment, but showed significant differences after re-watering (Figure 7F).

### 2.7. Physiological and Biochemical Properties in Transgenic Arabidopsis under Salt and Drought Treatments

**Figure 8:**
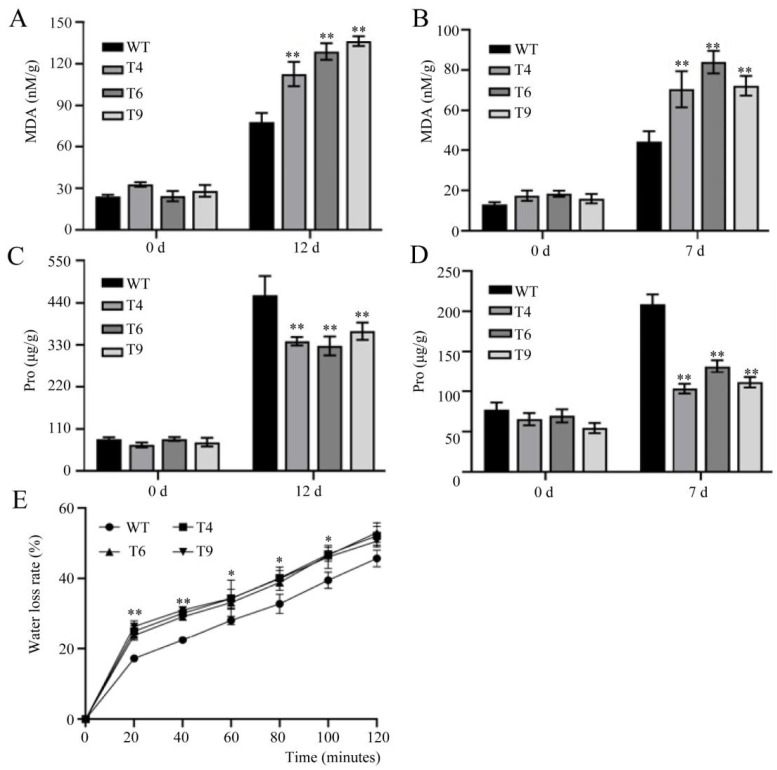
MDA, Pro (proline) content and water loss rate in *FtMYB22* transgenic *Arabidopsis* (T4, T6, and T9) lines and WT lines under stress treatments. (**A**) MDA content in transgenic lines under water deficit treatment. (**B**) MDA content in transgenic lines under salt treatment. (**C**) Proline content in transgenic lines under water deficit treatment. (**D**) Proline content in transgenic lines under salt treatment. (**E**) Water loss rate in isolated leaves of *FtMYB22* transgenic *Arabidopsis*. WT, wild-type. Data are shown as mean ± SD derived from three independent experiments. Error bars represent standard errors. ** means extremely significant difference at *p* < 0.01 level, * means significant difference at *p* < 0.05 level.

Physiological and biochemical properties are often analyzed as important indices for assessing plant resistance. Here, the levels of MDA and proline were first examined under normal and stress conditions. Under normal conditions, the MDA content of *FtMYB22* overexpressed *Arabidopsis* was not significantly different from that of the WT. However, the MDA accumulation of the transgenic lines was lower than that of the WTs when the plants were subjected to water deficiency and salt stress, which suggests that the transgenic plants may have suffered more severe membrane damage (Figure 8A,B). Moreover, transgenic plants has lower levels of proline after stress treatments (Figure 8C,D). Water holding capacity is a key index of plant stress tolerance. Here it was shown that the transgenic lines had a higher water loss rate than the WTs at each time point (Figure 8E), which suggests that the overexpression of *FtMYB22* resulted in reduced water-holding capacity in transgenic plants.

**Figure 9:**
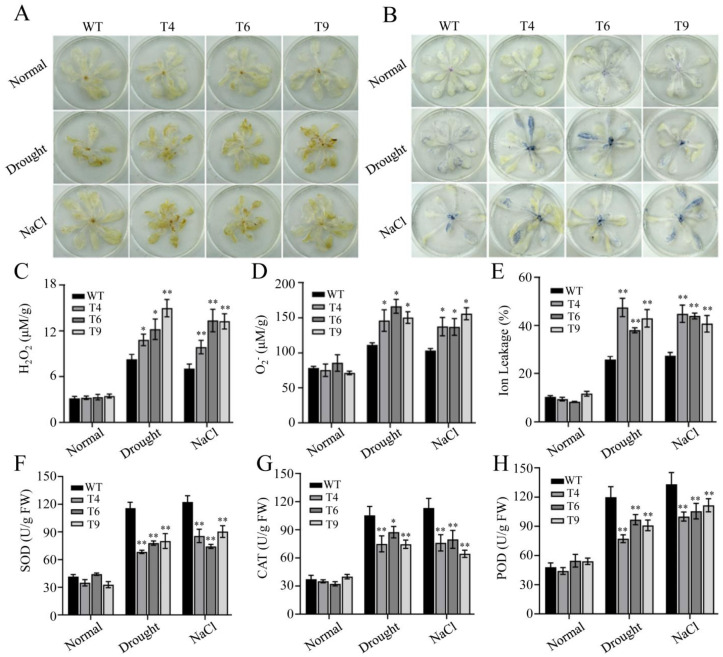
Peroxide staining and the peroxidase activity in *FtMYB22* transgenic *Arabidopsis* (T4, T6, and T9) lines and WT lines under stress treatments as indicated. (**A**) DAB staining. (**B**) NBT staining. (**C**) The content of H_2_O_2_. (**D**) The content of O_2_^−^. (**E**) Ion leakage. (**F**–**H**) Contents of SOD (**F**), CAT (**G**), and POD (**H**) were calculated from triple experiments. SOD, superoxide; CAT, catalase; POD, peroxidase. Normal: under normal conditions. WT, wild-type. Data are presented as mean ± SD. Three independent experiments were performed. Error bars indicate standard errors. ** means extremely significant difference at *p* < 0.01 level, * means significant difference at *p* < 0.05 level.

Adverse stress is often accompanied by the formation of ROS (reactive oxygen species), which disrupts membranes and macromolecules. DAB (diaminobenzidine) and NBT (nitroblue tetrazolium) staining were chosen to evaluate the degree of oxidative stress in plants. Figure 9A,B show that the DAB and NBT staining of plants under normal conditions were not obviously different, but all three transgenic lines showed a darker yellow and blue color when plants were subjected to stress treatments, which indicates that the transgenic plants accumulated more H_2_O_2_ and O_2_^−^ than the WTs. Similar results were obtained quantitatively for H_2_O_2_ and O_2_^−^ accumulation. After treatments, H_2_O_2_ and O_2_^−^ content increased in both the transgenic and WT plants, whereas the accumulation in the transgenic plants was significantly higher than that in the WTs (Figure 9C,D). Ion leakage (IL) also displayed a similar pattern to H_2_O_2_ and O_2_^−^ in all plants under the same conditions (Figure 9E). Furthermore, higher levels of H_2_O_2_ and O_2_^−^ in transgenic plants implied increased oxidative damage under water deficit and salt treatments, which prompted us to detect the activity of key antioxidant enzymes (SOD, superoxide dismutase, CAT, catalase, and POD, peroxidase). The activity of SOD, CAT, and POD in transgenic plants was significantly lower than in WTs under stress conditions (Figure 9F–H). These results were consistent with the drought and salt-tolerant phenotype of *FtMYB22* transgenic plants under stress conditions.

### 2.8. Overexpression of FtMYB22 Delays Stomatal Closure in Transgenic Arabidopsis under Stress Treatment

**Figure 10:**
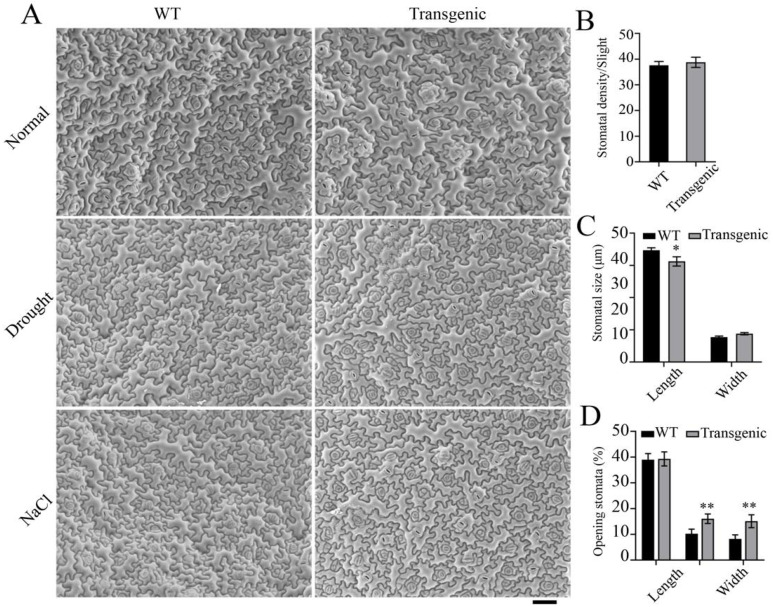
Stomatal phenotypes in *FtMYB22* transgenic *Arabidopsis* (T4, T6, and T9) lines and WT lines under stress treatments. (**A**) Stomatal observation. (**B**) Stomatal density per vision. (**C**) Length and width of stoma. (**D**) Stomatal opening rate. Normal: under normal conditions. WT, wild-type. Scale bar: 400 µm. Data are presented as mean and SD values of three independent experiments. Error bars indicate SD. ** means extremely significant difference at *p* < 0.01 level. * means significant difference at *p* < 0.05 level.

When plants suffer from abiotic stress, they usually adjust the stomata closure to respond. In order to study whether FtMYB22 has any effects on the stomata of transgenic plants, the stomatal number, size, and closure frequency of *FtMYB22* transgenic and WT plants were detected under normal and stress conditions (Figure 10). The results showed that there was no significant difference in the total number of stomata between transgenic and WT plants under normal conditions (Figure 10B). However, the overexpression of *FtMYB22* significantly reduced the stomatal length of transgenic plants by 10% compared with WTs, and did not affect stomatal width (Figure 10C). The switching proportion of stomata under different conditions was also counted. The results indicated that nearly 80% of stomata in all plants were open, and there was no significant difference between the transgenic and WT plants under normal conditions. However, under stress conditions, only about 20% of the stomata were open in WT plants, while more than 30% were open in transgenic plants (Figure 10D). The above results indicated that overexpression of *FtMYB22* significantly delayed the shutdown state of stomata in transgenic plants under water deficit and salt conditions.

### 2.9. Overexpression of FtMYB22 Reduces the Expression Levels of Stress-Response Genes

**Figure 11:**
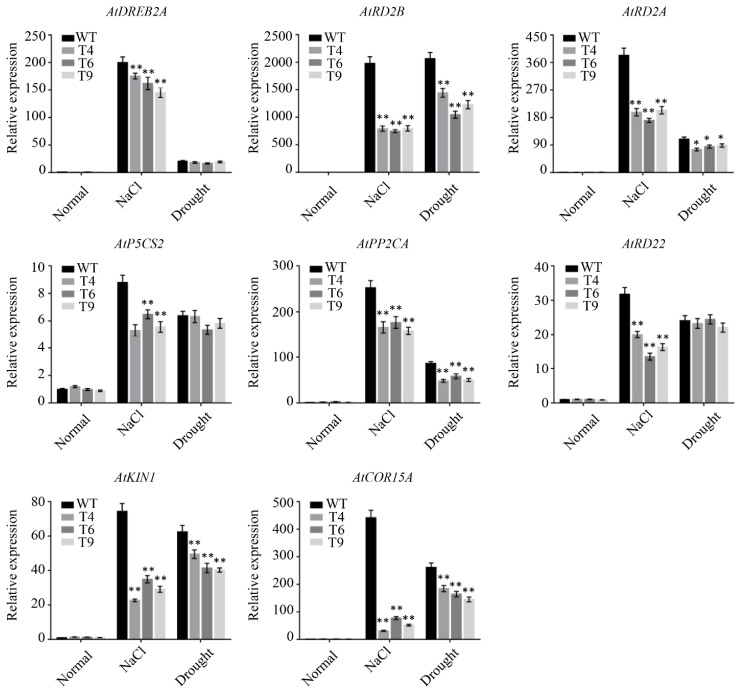
Expression levels of stress-associated genes following overproduction of FtMYB22 in *Arabidopsis* (T4, T6, and T9) lines and WT lines under stress treatments. Normal: under normal condition. WT, wild-type. Data are presented as mean and SD values of three independent experiments. Error bars indicate SD. ** means extremely significant difference at *p* < 0.01 level. * means significant difference at *p* < 0.05 level.

The expression of stress-associated genes was detected by qRT-PCR to determine whether these genes were regulated by *FtMYB22* (Figure 11). Under normal growth conditions, the expression levels of stress response genes in *FtMYB22* overexpression lines and WTs were almost the same. When salt stress was applied, these genes were down-regulated in *FtMYB22* overexpressing lines and WTs to various extents. Remarkably, among these genes, the expression levels of the ABA signaling genes (*AtRD29A*, *AtRD29B*, *AtPP2CA*, and *AtRD22*) were significantly lower in *Arabidopsis* over-expressing *FtMYB22* lines than in WTs after stress. Under water deficiency treatment, except for *AtDREB2A*, *AtP5CS2* and *AtRD22*, all other genes showed similar trends to those under salt stress. Overall, the reduced expression of stress-associated genes in *FtMYB22* overexpression lines was more pronounced than in WTs. These results suggest that the reduced water deficit or salt tolerance of *FtMYB22* transgenic plants may be due to the inhibition of the ABA signaling pathway and the expression of corresponding stress genes.

### 2.10. FtMYB22 Enhances the Sensitivity of Transgenic Arabidopsis to ABA

The expression level of *FtMYB22* was significantly up-regulated after ABA treatment. Meanwhile, an ABRE element that could respond to ABA was also found in the *FtMYB22* promoter sequence. Therefore, in order to further clarify the relationship between ABA and *FtMYB22* expression, *ProFtMYB22* transgenic plants were treated with exogenous ABA. The GUS histochemical staining and *GUS* gene expression were detected before and after ABA treatment. The results showed that, after ABA treatment, the GUS staining degree and the expression level of *GUS* gene controlled by *ProFtMYB22* in transgenic plants were significantly higher than those of transgenic plants under normal conditions (Figure S5). Therefore, the above results assume that *FtMYB22* may be involved in plant responses to abiotic stress through an ABA-dependent pathway.

**Figure 12:**
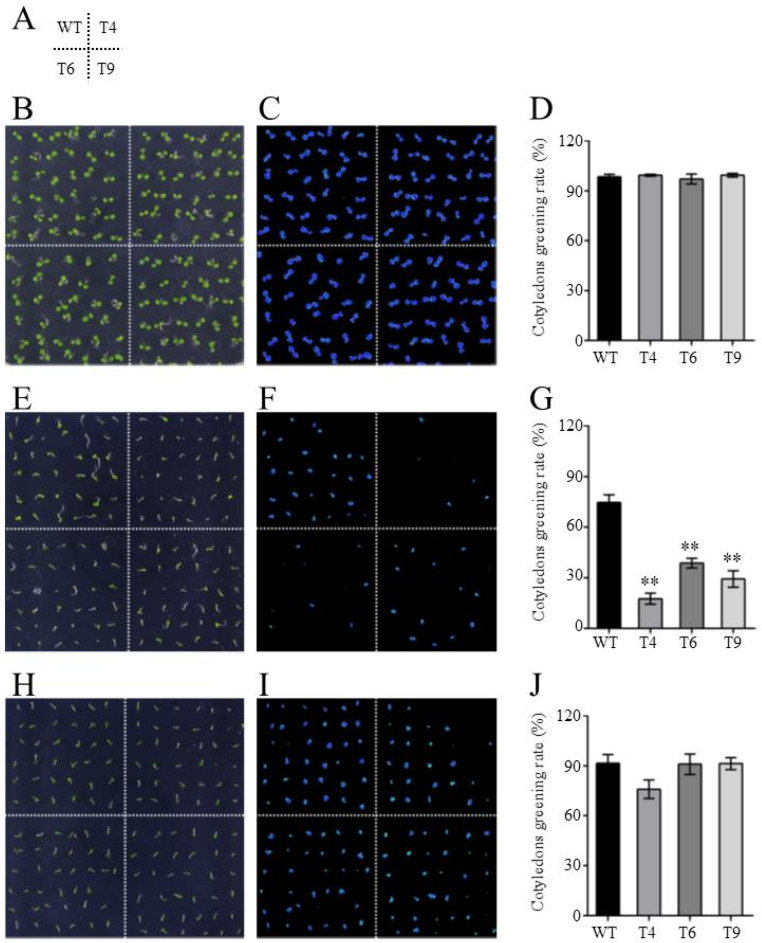
Effects of ABA treatment on germination rate of *FtMYB22* transgenic *Arabidopsis* (T4, T6, and T9) lines and WT lines. (**A**) The distribution of each line on the plate. (**B**–**D**) Normal conditions. (**E**–**G**) ABA treatment. (**H**–**J**) ABA and ABA synthesis inhibitors treatment. WT, wild-type. Data are presented as mean and SD values of three independent experiments. Error bars indicate SD. ** means extremely significant difference at *p* < 0.01 level.

To further study the relationship between ABA and *FtMYB22* function, the cotyledon greening rate and root length of transgenic plants overproducing FtMYB22 were determined under the ABA conditions. The cotyledon greening rate of transgenic plants was recorded in the whole growth process under treatment with concentrations of 1 μM of ABA. As shown in Figure 12D, under normal conditions, the cotyledon greening rate of all lines reached almost 80% after sowing for 3 days and more than 99% after 4 days of growing. Under the condition of 1 μM of ABA exogenous treatments, the germination rate of all lines was significantly inhibited and slowly germinated after 6 days. 10 days later, the cotyledon greening rate of the WTs reached 74.6% (Figure 12G), while the transgenic lines were only 17.6%, 38.8%, and 29.3% for T4, T6, and T9, respectively. Moreover, over the whole treatment period, the cotyledon greening rate of transgenic plants was significantly lower than that of the WTs. These results suggested that the overexpression of *FtMYB22* may affect the synthesis of ABA in transgenic plants. Hence, sodium tungstate, an inhibitor of ABA synthesis, was chosen to simulate the blocked ABA synthesis in plants. Figure 12J shows that, after adding the inhibitors, although the germination rate of all plants was affected by exogenous ABA to some extent, there was no significant difference between transgenic and WT plants during the whole growth process.

**Figure 13:**
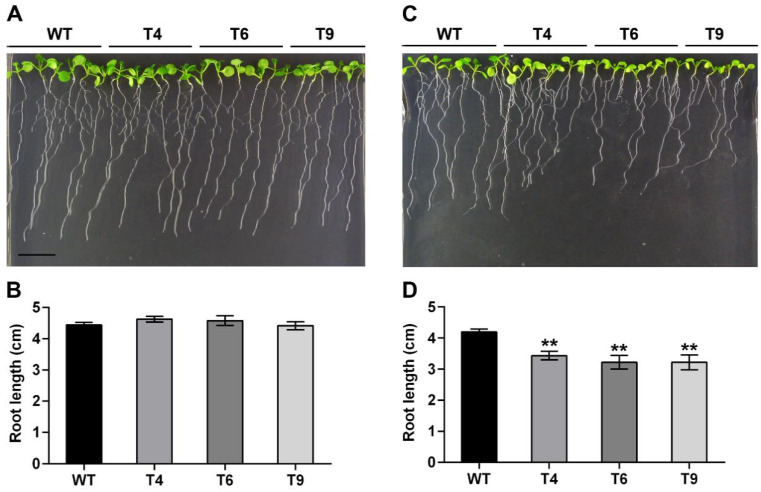
Effects of ABA stress on root length of *FtMYB22* transgenic *Arabidopsis* (T4, T6, and T9) lines and WT lines. (**A**,**B**) Root length under normal conditions. (**C**,**D**) Root length under ABA condition. WT, wild-type. Data are presented as mean and SD values of three independent experiments. Error bars indicate SD. ** means extremely significant difference at *p* < 0.01 level.

In addition, the root development of transgenic plants under ABA stress was also determined. *FtMYB22* transgenic and WT seeds were vertically cultured on 1/2 MS medium for 3 days, and then the seedlings with the same root lengths were selected and transferred to the medium containing ABA. As shown in Figure 13C, there was no significant difference in the root lengths of all lines under normal conditions. But the root growth of all lines was inhibited under the ABA condition, and the inhibition degree of *FtMYB22* transgenic lines was significantly stronger than that of WTs. Data showed that the root length of the three transgenic lines was only 81.7%, 76.6%, and 76.5% of WT root length under the ABA condition for T4, T6, and T9, respectively (Figure 13D).

### 2.11. FtMYB22 Interacts with Cis-Responsive Element ABRE

As an ABA response element, the ABRE is a ubiquitous element in almost all gene promoters that can be induced by ABA. The above results showed that FtMYB22 significantly affected the expression level of several genes responding to ABA signals. Hence, we speculated as to whether this element could also specifically bind to FtMYB22 while responding to ABA, thereby regulating the expression level of genes at the downstream. A yeast one-hybrid assay was performed to verify the possibility of binding between FtMYB22 and ABRE. First of all, different concentrations of 3-AT were used to screen the best concentration to inhibit the production of background His. A total of 80 mM was selected as the optimal 3-AT concentration for His inhibition (Figure S6).

**Figure 14:**
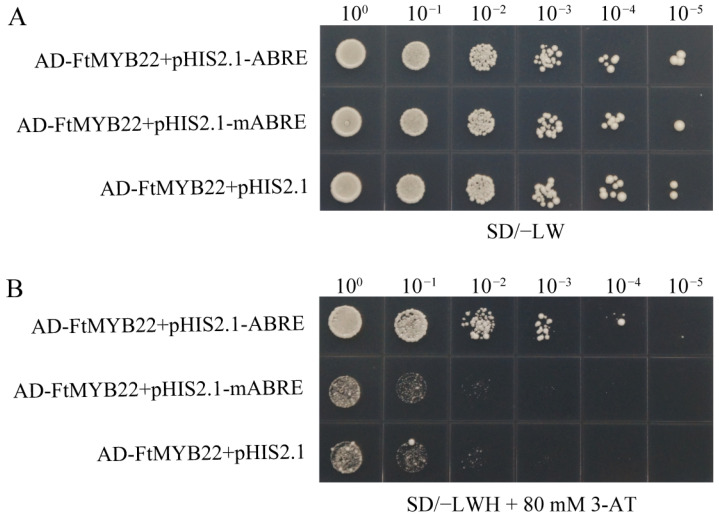
Yeast single hybridization of FtMYB22 with ABRE binding elements. (**A**) Growth phenotype of yeasts on SD/−LW (SD/−Trp/−Leu) medium. (**B**) Growth phenotype of yeast on SD/−LWH (SD/−Trp/−Leu/−His) + 3-AT medium. Yeast cells were co-transformed with the plasmids as indicated. The yeast cells co-transformed plasmids were serially diluted by 10, 100, 1000, 10,000 and 100,000 folds.

On SD/-Trp/-Leu (SD/-LW) medium, the positive yeasts respectively co-transformed with the plasmids of pGADT7-FtMYB22 + pHIS2.1-ABRE, pGADT7-FtMYB22 + mABRE, and pGADT7-FtMYB22 + pHIS2.1 all grew normally (Figure 14A). With a series of dilutions, the growth of bacterial colonies also showed a certain gradient, indicating that all plasmids had been successfully introduced into yeast, and the growth status of each colony was similar. However, on SD/−Trp/−Leu/−His (SD/−LWH) medium containing 80 mM of 3-AT, the transformants of all the negative control combinations were significantly repressed, and only the yeast strain with pGADT7-FtMYB22 + pHIS2.1-ABRE could grow normally (Figure 14B). These results indicate that FtMYB22 can specifically interact with the ABRE element.

### 2.12. FtMYB22 Is Involved in ABA-Dependenct Pathway through the Interaction with RCARs

ABA receptors are the extreme upstream regulators of the ABA signaling pathway, and are responsible for both recognizing the ABA signaling and initiating the primary process of signal transduction. The above results showed that the overexpression of *FtMYB22* enhanced the sensitivity of transgenic *Arabidopsis* to ABA, so this study investigated whether *FtMYB22* has an effect on the function of ABA receptor proteins. Therefore, the interaction between *FtMYB22* and ABA receptor RCAR (Regulatory Component of ABA Receptor) in *Arabidopsis* was studied. Firstly, the self-activation of all RCAR proteins was verified. Results showed that the combination of these plasmids could not activate the reporter genes downstream of the yeast system, indicating that no self-activation phenomenon occurred (Figure S7).

**Figure 15:**
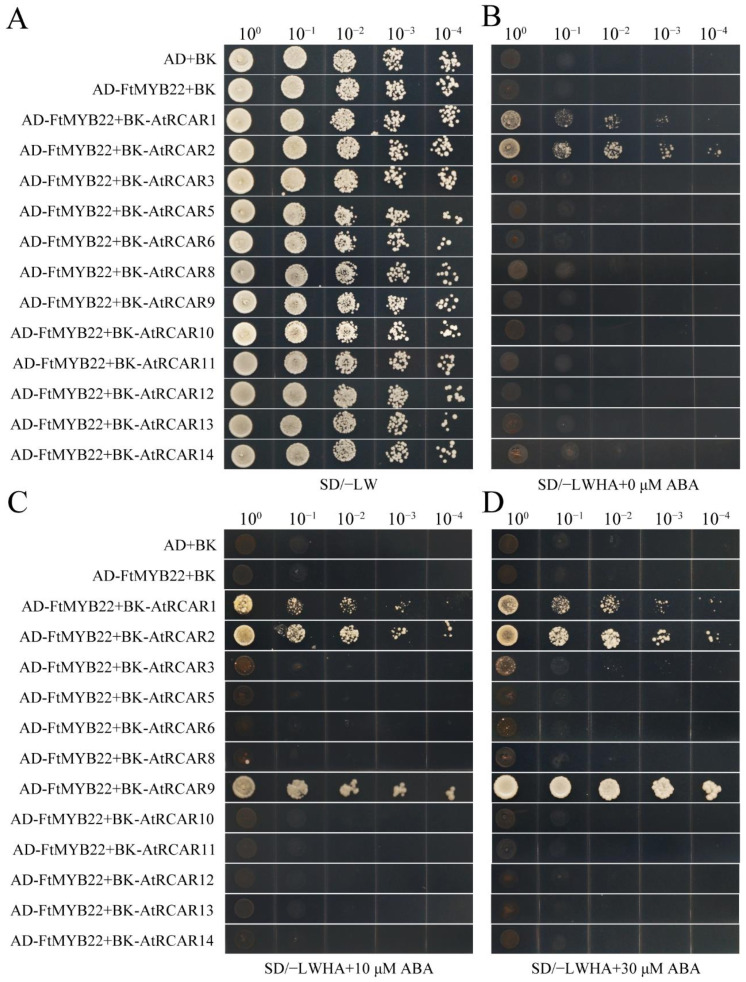
Molecular interactions between FtMYB22 and AtRCARs by Y2H. (**A**,**B**) The yeast cells carrying the plasmids as indicated were cultured on SD/−Leu/−Trp medium (SD/−LW) and SD/−Trp/−Leu/−His/−Ade medium (SD/−LWHA). (**C**,**D**) The yeast cells carrying the plasmids as indicated were cultured on SD/−Trp/−Leu/−His/−Ade medium containing 10 μM and 30 μM of ABA. The yeast cells containing recombinant plasmid as were serially diluted by 1, 10, 100, 1000, and 10,000 folds.

FtMYB22 and AtRCARs were co-transformed into the yeast cell AH109, and the growth status for each was observed in SD/−Trp/−Leu and in SD/−Trp/−Leu/−His/−Ade medium. Results showed that all transformed cells could grow normally in SD/−Trp/−Leu medium, indicating that the plasmids had been transferred into yeast, and the growth activity of different combinations was similar (Figure 15A). However, only the transformants of FtMYB22 and AtRCAR1/2 could grow normally on SD/−Trp/−Leu/−His/−Ade medium, which suggests that FtMYB22 could interact with AtRCAR1 and AtRCAR2 (Figure 15B). Moreover, in order to further understand whether the exogenous ABA would affect the interaction between FtMYB22 and AtRCARs, 10 μM and 30 μM of ABA were respectively added to SD/−Trp/−Leu/−His/−Ade medium. With the stimulation of exogenous ABA, the interaction between FtMYB22 and AtRCAR1/2 was slightly enhanced. Notably, FtMYB22 did not interact with AtRCAR9 under the normal condition. However, there was a strong interaction following the stimulation by ABA, and this interaction ability under the condition of treatment with 30 μM of ABA was significantly stronger than that of 10 μM of ABA (Figure 15C,D). It highlighted that the interaction between FtMYB22 and AtRCAR9 depended on the stimulation by ABA and in a dose-dependent way.

## 3. Discussion

### 3.1. FtMYB22 Reduces the Tolerance of Transgenic Plants to Water Deficit and Salt Stress

To date, knowledge about stress-associated R2R3-MYB TFs derives from research on model plants and major crops, such as *Arabidopsis*, rice, and wheat [21,22]. Tartary buckwheat has stronger tolerance to abiotic stress than other crops; however, only a few stress-related R2R3-MYB TFs have been studied. Therefore, deeply investigating these TFs and their regulatory mechanism has great significance in improving the abiotic stress resistance of Tartary buckwheat. In this study, an R2R3-MYB transcription factor *FtMYB22* induced by water deficiency and salt was identified from Tartary buckwheat. When compared with other reported R2R3-MYB TFs in Tartary buckwheat, *FtMYB22* showed unique response characteristics under a variety of stress conditions. In general, at the early stage of stress response, the gene expression is induced rapidly and decreases after reaching the highest level within a few hours [23]. However, upon water deficit and salt treatments, the *FtMYB22* expression was induced immediately and maintained at a very high level for a long time, which might have correlated with the strong basic stress resistance of Tartary buckwheat. Analysis of the *FtMYB22* promoter showed that MBS element was the core element in response to water deficit and salt treatments (Figure 3). These results suggest that *FtMYB22* may play an important role in tolerating water deficiency and salt stress.

Previous studies have shown that the overexpression of stress-related MYB TFs can change the stress tolerance of plants. For example, *CmMYB2* promoted the water deficiency tolerance and salt tolerance of transgenic *Arabidopsis* following induction by ABA, water deficit treatment, and salt treatment [24]. Similarly, the overexpression of *AtMYB44* also enhanced the tolerance of WT plants to water deficiency and salt stress [11]. On the contrary, the deletion of salt-induced *AtMYB73* enhanced the salt tolerance of *Arabidopsis*, indicating that *AtMYB73* negatively regulates salt stress [16]. The heterologous expression of *FtMYB10* significantly reduced the water deficit and salt tolerance of transgenic *Arabidopsis* [25]. In this study, the overexpression of *FtMYB22* significantly decreased the cotyledon greening rate (Figure 5), root length (Figure 6), and survival rate (Figure 7) of transgenic *Arabidopsis* lines under water deficit and salt conditions. Similar to *AtMYB73* and *FtMYB10*, *FtMYB22* may also act as a negative regulator of plant responses to water deficit and salt stress.

### 3.2. Physiological and Biochemical Responses of FtMYB22-Overexpressed Arabidopsis under Drought and Salt Stress

When plants are subject to abiotic stresses, such as water deficit and salt stress, some physiological indicators respond quickly to improve plant survival in these extreme environmental conditions. Hence, the physiological indices related to water deficiency and salt stress can be used as rapid and accurate reporters to evaluate the resistance ability in plants. IL, an important physiological parameter, is usually used to reflect the degree of cell membrane damage [26]. In this study, the IL of plants overproducing FtMYB22 was significantly higher than that of WTs (Figure 9E), indicating that the overexpression of *FtMYB22* destroyed the integrity of the cell membrane and reduced the tolerance to water deficit and salt stress. Abiotic stresses can also cause lipid peroxidation, resulting in a considerable accumulation of MDA [26]. The data here showed that the MDA content of *Arabidopsis* lines overproducing FtMYB22 was higher than that of WTs under salt and water deficit stress (Figure 8A,B), which illustrated that *FtMYB22* could activate oxidative stress and cause more serious oxidative damage. In addition, proline could counteract the effects of osmotic pressure by scavenging free radicals and buffering cell redox [27]. In this study, *FtMYB22*-overexpressed *Arabidopsis* lines accumulated less proline than WTs under water deficit and salt stress (Figure 8C,D), which suggests that the decrease in proline accumulation might also be one of the reasons why transgenic plants showed weaker water deficit and salt tolerance. It has been shown that SOD, POD and CAT are important antioxidant enzymes for protecting plants from abiotic stress [28]. FtMYB22 overproduction increased H_2_O_2_ and O_2_^−^ and lowered SOD, POD and CAT activities in transgenic plants compared to WTs under stress (Figure 9), which further provided a basis for the role of FtMYB22 in reducing water deficit and salt resistance in plants. Altogether, the decrease in the water deficit and salt tolerances of *FtMYB22* transgenic plants was at least partly correlated with the increase in MDA content and IL, the decrease in proline and soluble sugar content, and the decrease in SOD, POD, and CAT activity.

In addition to the above physiological indices and enzyme activity, the decrease in tolerance to water deficiency and salt stress is also relevant to the expression of response genes to abiotic stress [29]. For example, *FtMYB10* overexpression inhibits the expression of stress response genes in transgenic *Arabidopsis*, thereby reducing the tolerance to water deficit and salt stress [25]. Similarly, in this study, the expression levels of *AtRD29A*, *AtP5CS2*, *AtRD29B*, *AtKIN1*, *AtPP2CA*, and *AtCOR15A* in plants overexpressing FtMYB22 were lower than those in WTs under water deficit and salt stress (Figure 11). It has been reported that the expression of *AtP5CS2* is positively correlated with proline accumulation, and the overexpression of *AtP5CS2* increases the resistance to abiotic stress [30]. Therefore, *FtMYB22* may respond to abiotic stress through down-regulating the expression of *AtP5CS2* to inhibit proline synthesis under abiotic stress. *FtMYB22* also regulates the expression level of other stress-related genes to cope with stress conditions, such as *AtRD29A*, *AtRD29B*, *AtKIN1*, and *AtCOR15A* [31]. However, under normal growth conditions, there was no significant difference in the expression of these abiotic stress-related genes between plants overexpressing *FtMYB22* and WTs. One possible explanation is that under stress conditions, other stress regulators are required to activate *FtMYB22*-dependent stress response genes. For example, the overexpression of *nNOS* in rice enhanced the expression of stress response genes under abiotic stress, while under normal conditions, transgenic lines and WTs showed similar gene expression levels [32].

### 3.3. FtMYB22 Participates in Plant Response to Abiotic Stress through ABA-Dependent Pathway

Hormones are the central regulatory factors in plant responses to abiotic stress. Among all plant hormones, the ABA signal pathway and JA signal pathway are the two most vital hormone regulatory pathways [33]. It has been suggested that the significant increase in ABA levels under water deficiency or salt stress is due to changes in gene expression and adaptive physiological responses. The expression levels of genes in the ABA synthesis pathway are up-regulated under water deficit stress, while they are also regulated by major transcription factor families, including bZIP, MYB, MYC, NAC, ERF, and DREB/CBF [34]. The overexpression of rice *OsNAC9* significantly improved the drought resistance of its transgenic plants. The results of qRT-PCR showed that the expression level of *NCED*, the key and rate-limiting gene in ABA synthesis pathway, increased by more than 20 times in transgenic plants compared to WTs [35]. As the most abundant family of TFs in plants, MYB is regulated by the ABA hormone, which mediates the plant response to stress through an ABA-dependent pathway. *AtMYB60*, *AtMYB44*, and *AtMYB15* in *Arabidopsis* lines have been shown to respond to water deficit and salt stress by ABA-mediated stomatal closure [36]. The overexpression of poplar R2R3-MYB TF *PtrSS* in *Arabidopsis* lines significantly improved the tolerance to salt stress and sensitivity to ABA [37]. Cherry PacMYBA, as an ABA-dependent TF, enhanced anthocyanin synthesis, pathogen resistance, and salt tolerance in plants [38].

In this study, the expression of *FtMYB22* was strongly induced by exogenous ABA, and its heterologous expression in *Arabidopsis* enhanced the sensitivity to ABA. The expression levels of several ABA-dependent marker genes—*AtABA1*, *AtABI3*, *AtABF3*, and *AtNCED9* were up-regulated under the induction of ABA, and the promoter regions of these genes also contained single/multiple *cis*-acting sequences, namely ABRE [39,40]. FtMYB22 can specifically bind to ABRE (Figure 14), which demonstrates that FtMYB22 may activate the expression of genes in the downstream of the ABA signal pathway and participate in the stress response process. In addition, this study also found that FtMYB22 interacted with SnRK2.3 proteins in *Arabidopsis* and Tartary buckwheat. It is well known that SnRK protein functions in the ABA signal transduction pathway. Under the presence of ABA, ABA forms a complex with its receptor RCAR and PP2C to activate SnRK2 protein activity. Subsequently, the activated SnRK2 can phosphorylate downstream substrate proteins, such as TFs, to promote the transcription of ABA response genes [41]. These results illustrate that FtMYB22 not only affects the accumulation of ABA, but also participates in the downstream signal transduction of ABA. When *FtMYB22* transgenic plants were treated with ABA, their growth was significantly inhibited. Therefore, it is speculated that the overexpression of *FtMYB22* gene not only affects ABA synthesis and downstream signal transduction, but also changes the perception of extracellular ABA signal in plant cells.

As a class of ABA receptor proteins, RCARs undertake the mission of recognizing the ABA signals and initiating the primary process of signal transduction [41]. For example, CARK1 regulates the ABA signal pathway by phosphorylating RCAR3/RCAR11, which enhances the drought response in plants after their overexpression in *Arabidopsis* [42]. RIFP1, as the adapter subunit of SCF ubiquitin ligase complex, negatively regulates the RCAR3-mediated ABA signal pathway by promoting RCAR3 degradation [43]. This highlights that RCARs protein plays an indispensable role in the function of ABA pathway-dependent genes. In this study, it was shown that the interaction between FtMYB22 and AtRCAR1/2 in *Arabidopsis* was stronger when exogenous ABA was added. Additionally, there was a strong interaction between FtMYB22 and AtRCAR9 when exogenous ABA was added, which did not interact with each other under normal conditions (Figure 15). Therefore, FtMYB22 possibly participates in plant responses to abiotic stress by affecting the formation or stability of the RCAR-ABA-PP2C complex. Except for the ABA signal pathway, the jasmonic acid signal pathway is also another hormonal pathway that responds to stress in plants. However, this study revealed that the overexpression of *FtMYB22* did not affect the response of transgenic *Arabidopsis* to methyl jasmonate. The above results suggest that *FtMYB22* may mediate plant responses to abiotic stress through the ABA-dependent pathway in three aspects: cell perception of the ABA signal, participation in ABA accumulation and downstream ABA signal transduction, and independence from the jasmonic acid signal pathway.

## 4. Materials and Methods

### 4.1. Plant Materials and Growth Conditions

Tartary buckwheat seeds were given by Prof. Anhu Wang of Xichang College in Xichang, Sichuan, China, and were grown in the experimental field at Sichuan Agricultural University in Ya’an, China. *Arabidopsis thaliana* ecotype Columbia-0 (wild-type, WT) were used for the transformation of *FtMYB22*, and were kept in an artificial climate chamber at 25 °C, with 60% relative humidity, and 250 µmol photons m^−2^·s^−1^ light intensity, with a different photoperiod (16 h/8 h light/dark for *Arabidopsis*).

### 4.2. DNA, RNA Extraction and qRT-PCR

The total genomic DNA of Tartary buckwheat were extracted using the Plant Genomic DNA Kit (TIANGEN, Beijing, China) and the total RNA of Tartary buckwheat and the transgenic plants was extracted using the RNAout Kit (TIANGEN, Beijing, China). A Revert Aid First Strand cDNA Synthesis kit (Takara, Dalian, China) was used to synthesize the cDNA. The quantitative real-time PCR (qRT-PCR) experiments were conducted using SYBR^®^ Premix Ex Taq™ (Tli RNaseH Plus) (Takara Bio Inc., Dalian, China) on a Bio-Rad CFX96™ Real-Time system (Bio-Rad, Hercules, CA, USA) following the manufacturer’s instructions. FtH3 and β-actin were used as internal references in Tartary buckwheat and *Arabidopsis*, respectively. The expression of six maker genes from *Arabidopsis* was determined, including *AtDREB2A*, *AtRD29B*, *AtRD29A*, *AtP5CS2*, *AtPP2CA*, *AtRD22*, *AtKIN1*, and *AtCOR15A*. All primers for PCR are listed in Table S1. The relative expression levels of the genes were calculated using the 2^−ΔΔCT^ method as described previously [44].

### 4.3. Isolation and Characterization of FtMYB22

In the previous study, a MYB TF gene, *FtMYB22*, was cloned from Tartary buckwheat [18]. Herein, the gene sequence was analyzed using the National Center for Biotechnology Information (NCBI) database (https://www.ncbi.nlm.nih.gov/, accessed on 20 August 2020). Multiple amino acid sequence alignments were performed using ClustalX7.0 software [45] and Weblogo (http://weblogo.berkeley.edu/logo.cgi, accessed on 25 August 2020). A phylogenetic tree between FtMYB22 and related proteins from other plant species was created using Geneious Prime2020.1 software [46].

To investigate the subcellular location of *FtMYB22*, the open reading frame (ORF) of *FtMYB22* was amplified and fused into the N-terminus of primary HBT95-*GFP* vectors under the control of *CaMV 35S* promoter, resulting in the *FtMYB22*-*GFP* construct. Recombinant plasmids *FtMYB22-GFP* and HBT95-*GFP* (used as negative controls), and *35S-RFP-NLS* (used as a nuclear marker) were separately introduced into the *Arabidopsis* protoplasts as previously described [47]. The transformed protoplasts were incubated at 22 °C for 24 h in darkness, and fluorescent images were captured with a confocal laser scanning microscope. (Leica DM IRBE, Berlin, Germany). The chloroplast autofluorescence images were obtained using the same microscope.

For the transcriptional activity assay, the coding region of *FtMYB22* was amplified and introduced into the primary *pBridge* vectors that contained the GAL4 DNA-binding domain, resulting in the *pBridge-FtMYB22* construct. Subsequently, the *pBridge-FtMYB22* and *pBridge-pGAL4* vectors (used as positive controls), and the *pBridge* vector (used as a nuclear marker) were transformed into the yeast (*Saccharomyces cerevisiae*) stain AH109. The transformed yeast stains were cultured on synthetic defined (SD) medium lacking tryptophan (Trp) and histidine (His; SD/-Trp/-His) at 30 °C for 3 days.

### 4.4. Cloning and Activity Analysis of the FtMYB22 Promoter

The promoter sequence (~2000 bp) of *FtMYB22*, named *ProFtMYB22*, was amplified from the genomic DNA of Tartary buckwheat using the specific primers *ProFtMYB22*-F and *ProFtMYB22*-R according to the Tartary buckwheat genome database [48]. The bioinformatic analysis of the promoter sequence was performed using the online tools Neural Network Promoter Predication (http://www.fruitfly.org/seq_tools/promoter.html, accessed on 20 March 2021) and PlantCARE (http://bioinformatics.psb.ugent.be/webtools/plantcare/html/, accessed on 25 March 2021). The functional domains, transcriptional start sites, and regulatory elements of the *FtMYB22* were predicted.

Based on the above prediction, the *FtMYB22* promoter was truncated in terms of the position of stress response elements, named *ProFtMYB22, ProFtMYB22-1*, and *ProFtMYB22-2*. The three promoter sequences were cloned into the upstream of the *glucuronidase (GUS)* gene in the *pBI101-GUS* vector to obtain the *ProFtMYB22-GUS*, *ProFtMYB22-1-GUS*, and *ProFtMYB22-2-GUS* constructs, respectively. The empty pBI101-*GUS* vector (negative control), *35S-GUS* (positive control), *ProFtMYB22-GUS*, *ProFtMYB22-1-GUS*, and *ProFtMYB22-2-GUS* were purified and introduced into the *Arabidopsis* (Col-0) by the *Agrobacterium tumefaciens* strain GV3101 using the floral dip method. Transgenic seedlings were screened on half Murashige and Skoog (1/2 MS) medium supplemented with kanamycin (50 mg/L). The resistant seedlings were transferred to pots for PCR and GUS staining.

For the abiotic stress assay, two-week-old transgenic seedlings were transferred to liquid 1/2 MS medium solution supplemented with Abscisic acid (ABA, 1 μM), mannitol (150 mM), or Sodium Chloride (NaCl, 150 mM). All samples were harvested at 0 h and 6 h from the corresponding treatments. Subsequently, half of these samples were frozen with liquid nitrogen and stored at −80 °C to extract RNA for *GUS* gene expression analysis. The rest of the seedlings were used for the histochemical analysis of GUS activity of *ProFtMYB22/-1/-2*.

### 4.5. Stress Tolerance Studies in Yeast with FtMYB22

Yeasts have been shown to be an effective system that can be used to evaluate the tolerance of TF genes, such as *EsDREB2B* [49] and *MuNAC* [50]. The full ORF of the *FtMYB22* was cloned and introduced into the *pYES2* vector (Invitrogen, Carlsbad, CA, USA) with primers *pYES2*-*FtMYB22*-F and *pYES2*-*FtMYB22*-R. Then, the *pYES2*-*FtMYB22* and empty *pYES2* (control) plasmids were transformed into yeast strain INVSc1 (Invitrogen, Carlsbad, CA, USA) using a lithium acetate procedure. The transformants were selected by their growth on uracil-deficient synthetic complete (SC/−Ura) medium with 2% (*w/v*) glucose at 30 °C. The positive colonies were identified by qRT-PCR. For the stress assay, transformed yeast cells were incubated in SC/-Ura liquid medium containing 2% (*w/v*) glucose at 30 °C. Upon incubation overnight, the sample densities were adjusted to OD600 ≈ 0.4 in 10 mL of induction medium (SC/−Ura medium supplemented with 2% *w/v* galactose), and then cultured for 36 h to promote *FtMYB22* gene expression. After incubation, the yeast cell densities were diluted to contain an equal number of cells (OD_600_ ≈ 1) in 200 μL solutions with different chemicals for the abiotic stress assays. For water deficit and salt stress, the yeast cells were cultured with 15% (*w/v*) PEG600 or 150 mM NaCl at 30 °C for 24 h, respectively. For cold stress, the yeast cells were cultured at −20 °C for 24 h. For UV-B stress, the yeast cells were cultured in a growth chamber with UV-B illumination (302 nm wavelength, 0.1 mW/cm^2^) for 24 h. For the control, an equal number of yeast cells were cultured at 30 °C for 24 h. After treatments, these samples were diluted by 10-fold (1, 10^−1^, 10^−2^, 10^−3^, 10^−4^, and 10^−5^) with sterile water, then 2 μL of cell solution was spread on SC/-Ura medium and incubated at 30 °C for 3 days to observe their growth performance.

### 4.6. The Generation of Transgenic Arabidopsis

The *FtMYB22* ORF was amplified and constructed into the plant expression vector *pCAMBIA1305-35S*, which resulted in *pCAMBIA1305-35S-FtMYB22*. Then, the *pCAMBIA1305-35S-FtMYB22* vector was transformed into wild-type *Arabidopsis* (WT) plants by Agrobacterium-mediated floral dip methods. The transgenic seedlings were screened on the 1/2 MS medium containing hygromycin (50 mg/L), and the resistant seedlings were transferred to the pots for growth. Lastly, positive transgenic lines were continually validated by PCR, and T3 homozygous lines over-expressing *FtNAC22* were selected for further analysis.

### 4.7. Abiotic Treatments of Transgenic Arabidopsis

For the osmotic stress assay of transgenic *Arabidopsis*, the seeds of transgenic and WT plants were surface-sterilized in 70% ethanol for 1 min for two times. Then the seeds were rinsed with sterilized water five times and dried at room temperature. Subsequently, the sterilized seeds were sown in the 1/2 MS medium containing ABA (1 μM), mannitol (150 mM), and NaCl (150 mM), respectively, and kept at 4 °C for vernalization. Two days later, these seeds were transferred into a light condition with 25 °C, 60% relative humidity, and 250 µmol photons m^−2^ s^−1^ light intensity. The cotyledon greening rate and root length were measured and repeated three times.

For the phenotype analysis assay, positive transgenic *Arabidopsis* lines and WT seeds were planted in pots filled with peat soil that were watered regularly for 3 weeks in an artificial climate chamber at 25 °C, 60% relative humidity, 250 µmol photons m^−2^ s^−1^ light intensity, and a 16 h light/8 h dark photoperiod. Then, half of the four-week-old plants were subjected to withholding water stress for 12 days and then re-watered. The rest of the plants were watered with 200 mM NaCl for 7 and 12 days. Meanwhile, the survival rate, water loss rate, fresh weight, proline (Pro), malondialdehyde (MDA), ion leakage (IL), superoxide anion (O_2_^−^), hydrogen peroxide (H_2_O_2_), diaminobenzidine (DAB) and nitroblue tetrazolium (NBT) staining were measured in the transgenic *Arabidopsis* plants and WTs after all the processing was completed as previously described [51]. To investigate whether stomatal pore closure was sensitive to ABA, the stomatal aperture was visualized according to the methods described previously [52,53].

### 4.8. Yeast Hybridization

For the yeast one-hybrid (Y1H) experiment, three repeat NACRS sequences (CGTA/CGTG/CGTA) were constructed in the form of interval TT into pHIS2.1 yeast one-hybrid vector. Subsequently, the recombinant plasmid pGADT7-*FtMYB22* (AD-FtMYB22) was transformed into yeast strain *Y1H187* containing the vector pHIS2.1-*NACRS*. Then, the interaction between DNA and protein was studied according to the Yeast Protocols Handbook (Takara Bio Inc., Dalian, China).

Yeast two-hybrid (Y2H) analysis was applied to determine whether the FtMYB22 protein could interact with AtRCARs. The CDS of the *FtMYB22* and *AtRCAR*s were inserted into pGADT7 (AD) and pGBKT7 (BK) vectors, respectively. The combination of AD-FtMYB22 + BK-AtRCARs was used as the experimental group, and the combinations of AD + BK and AD-FtMYB22 + BK were used as the negative control groups, which were each transformed into yeast AH109. The experiment was conducted according to Matchmaker^®^ Gold Yeast Two-Hybrid System operating manual (Takara Bio Inc., Dalian, China).

### 4.9. Statistical Analysis

The data obtained from three biological replicates were shown as the means ± SD. Statistical analysis of data was conducted using one-way analysis of variance (ANOVA), followed by Tukey’s comparison tests, or using the Student’s *t*-test in the SigmaPlot 10.1 software. Differences were considered statistically significant at *p* < 0.05 or *p* < 0.01.

## 5. Conclusions

**Figure 16:**
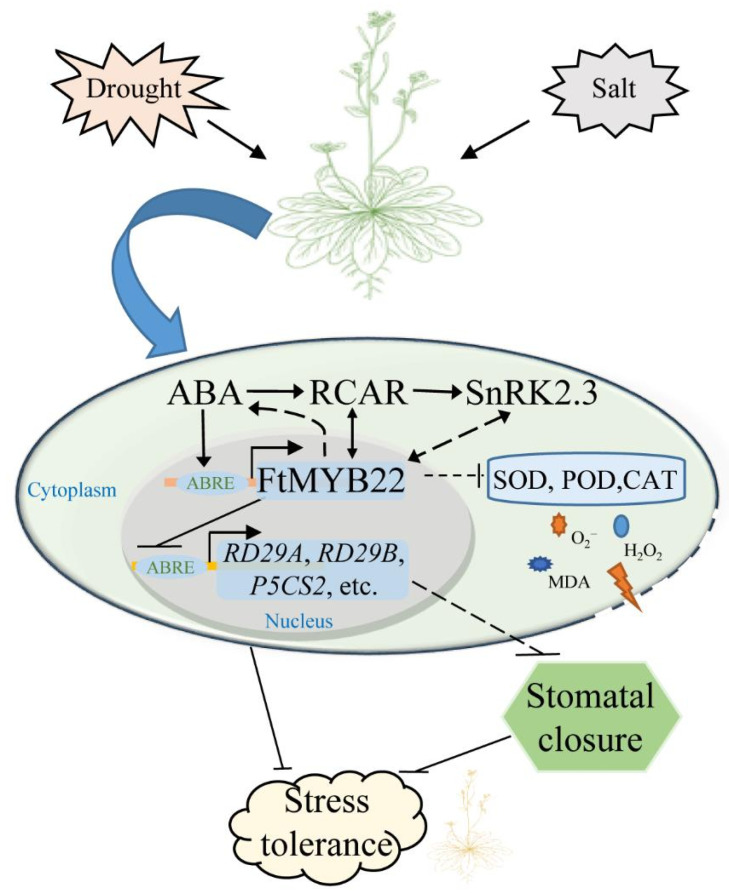
Hypothetical regulatory model of the FtMYB22 responding to abiotic stress. The solid lines represent confirmed conclusions and dashed lines represent hypothetic pathways.

This study demonstrated that FtMYB22 is a negative regulator in stress response. It showed that FtMYB22 inhibited a variety of physiological and biochemical reactions, repressed gene expression and stomatal closure in transgenic plants through the ABA-dependent pathway, and reduced the tolerance of transgenic *Arabidopsis* to water deficiency and salt stress (Figure 16). This study extended the study of R2R3-MYB transcription factors functioning in Tartary buckwheat stress resistance, and deepened the understanding of the complicated signal regulatory mechanism responding to abiotic stress in Tartary buckwheat. Thus, this study provided a new theoretical basis for the further cultivation of Tartary buckwheat germplasm resources with high resistance.

## Data Availability

Not applicable.

## Supplementary Materials

The following supporting information can be downloaded at: https://www.mdpi.com/article/10.3390/ijms232314549/s1.
