# Supplementary material for: A Novel R2R3-MYB Transcription Factor FtMYB22 Negatively Regulates Salt and Drought Stress through ABA-Dependent Pathway
Source: Int J Mol Sci. 2022 Nov 22;23(23):14549. doi: 10.3390/ijms232314549 (PMC9735685; doi:10.3390/ijms232314549)
Supplement: Supplementary file 1 [file ijms-23-14549-s001.zip › ijms-2010525-supplementary.pdf]

Supplementary Material

Supplementary Figures

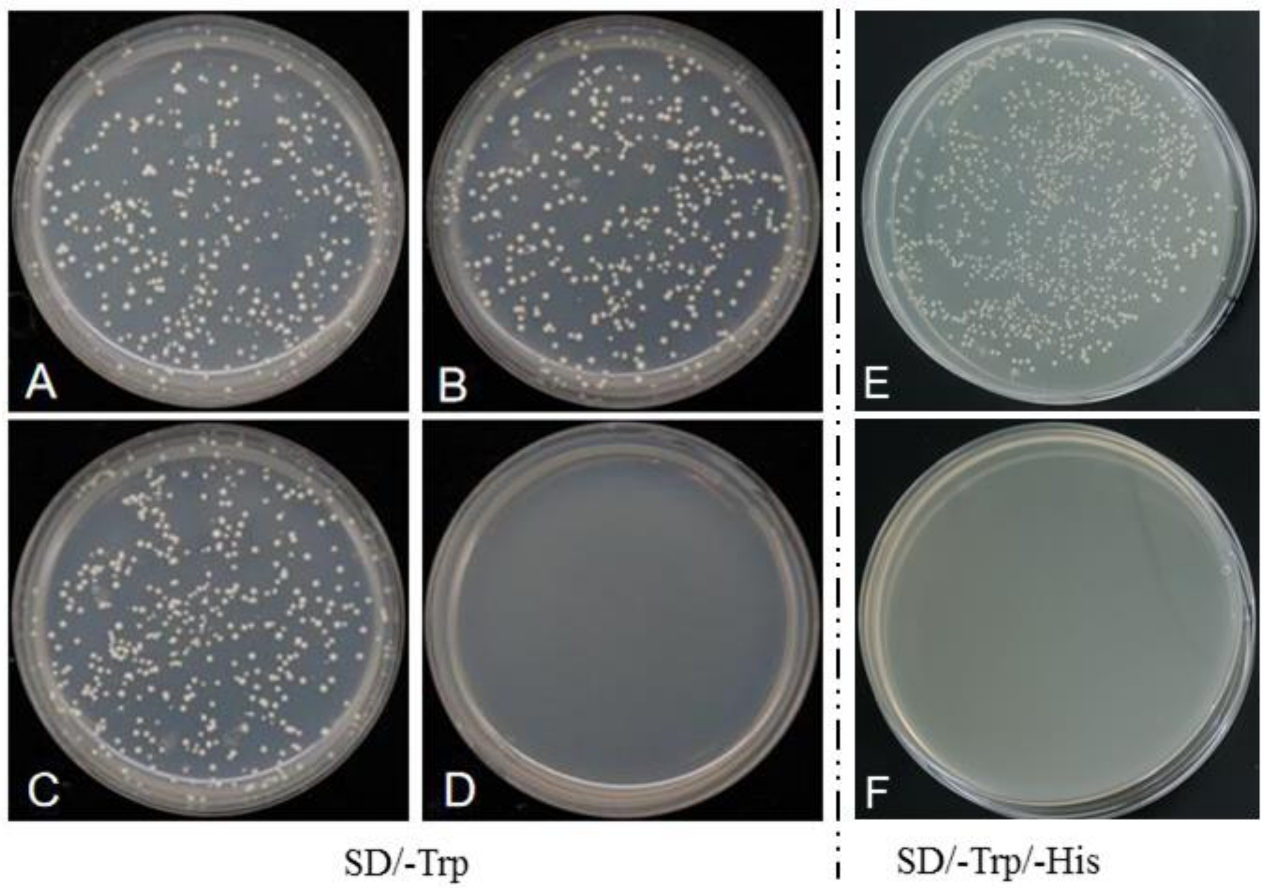

Figure S1 Transactivation assay of *FtMYB22* in yeast. (A)~(D): Plate screening of transformed yeast on SD/-Trp medium. A: AH109 cells transformed with pBridge-FtMYB22. (B): AH109 cells transformed with pBridge-GmMYBJ6 (positive control). (C): AH109 cells transformed with pBridge. (D): AH109 cells transformed with H_2O. (E)~(F): Plate screening of transformed yeast on SD/-Trp/-His medium. (E): AH109 cells transformed with pBridge-GmMYBJ6 (positive control). F: AH109 cells transformed with pBridge-FtMYB22.

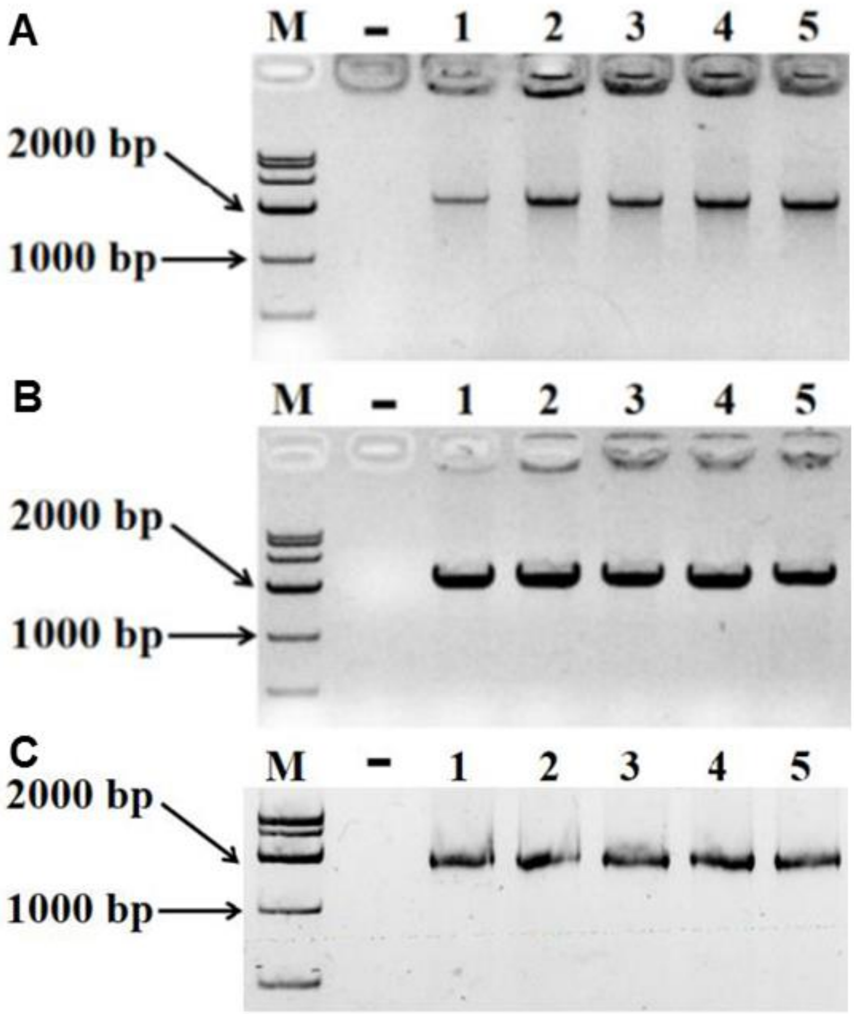

Figure S2. **PCR identification of *ProFtMYB22* transgenic *Arabidopsis*. (**A): PCR identification of *ProFtMYB22* transgenic *Arabidopsis*. (B): PCR identification of *ProFtMYB22-1* transgenic *Arabidopsis*. (C): PCR identification of *ProFtMYB22-2* transgenic *Arabidopsis*. M: DNA Marker. -: Negative control 1-5: PCR amplification products.

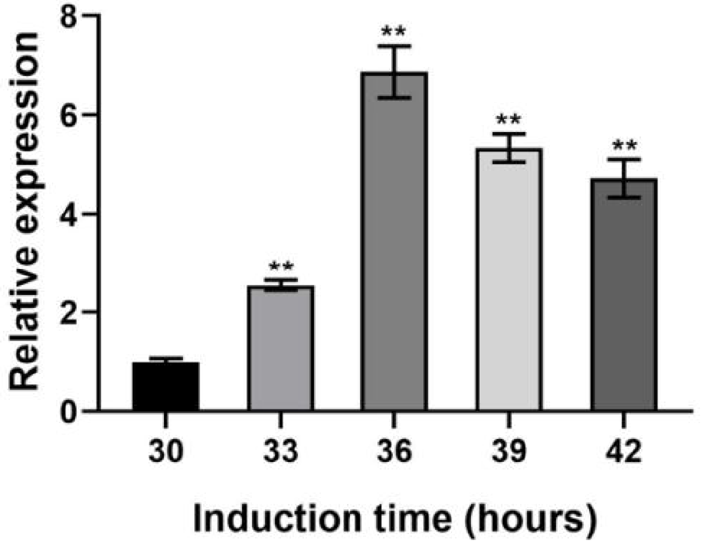

Figure S3 Expression analysis of *FtMYB22* at different induction time. ** means extremely significant difference at $P < 0.01$ level.

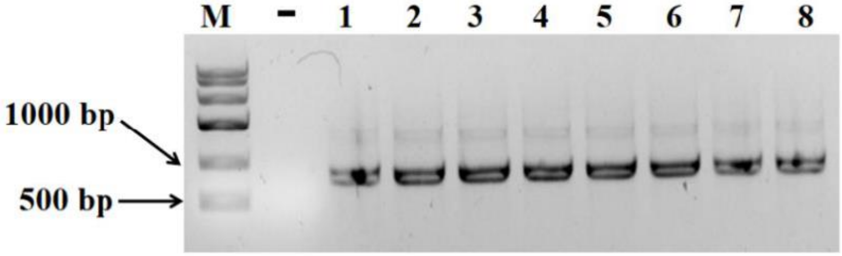

Figure S4 Positive identification of *FtMYB22* transgenic Arabidopsis. Identification of *FtMYB22* by PCR. M: DNA marker. -: Wild type. 1-8: Candidates of transgenic line. 3, 5, and 6 represented transgenic lines T4, T6, and T9, respectively.

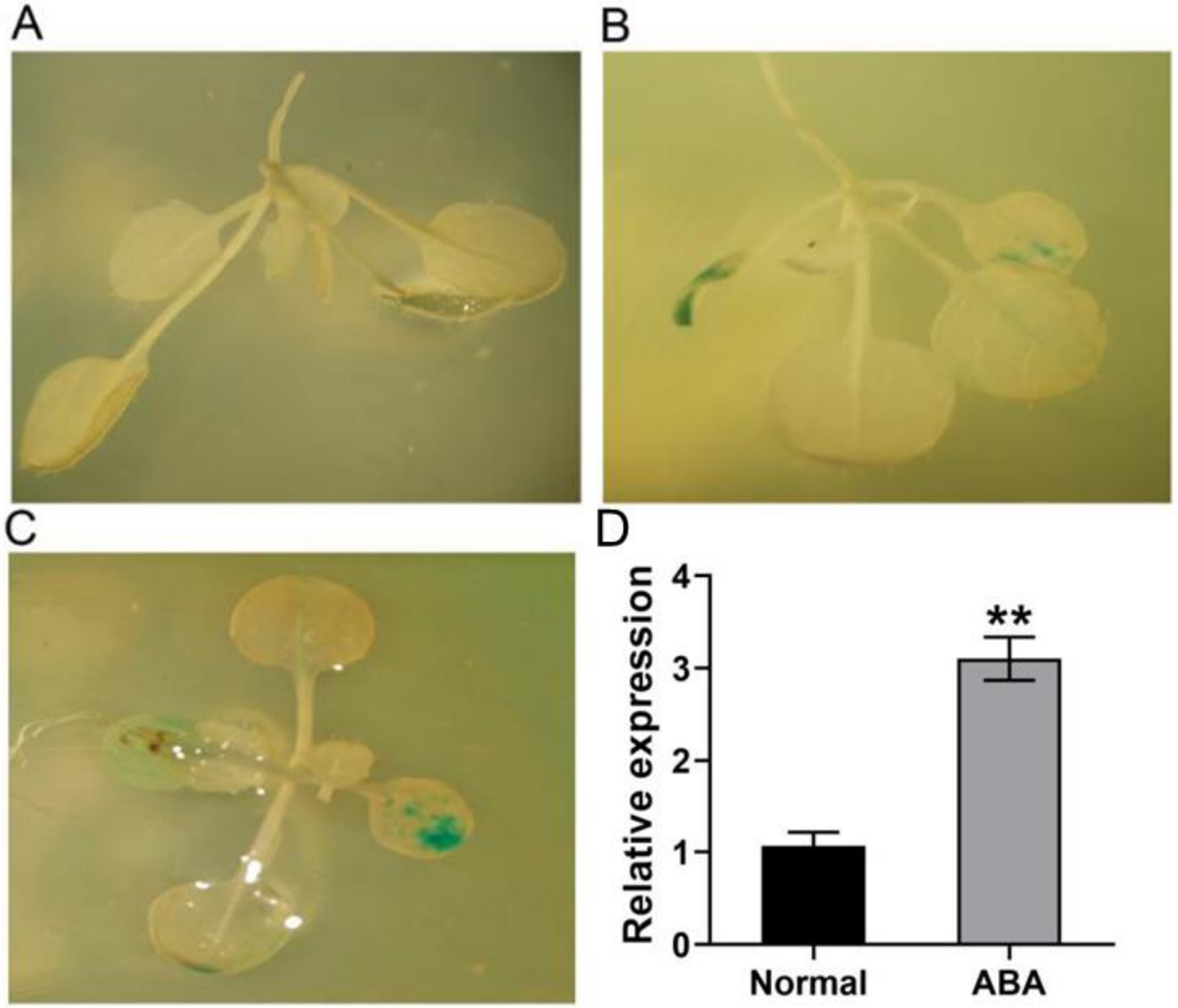

Figure S5 The expression of *GUS* gene in ProFtMYB22 transgenic plants under ABA treatment. **(**A): Negative control. (B): GUS staining of *ProFtMYB22* transgenic plants under normal condition. (C): GUS staining of *ProFtMYB22* transgenic plants under ABA treatment. (D): The expression of *GUS* gene in *ProFtMYB22* transgenic plants under ABA treatment ** means extremely significant difference at $P < 0.01$ level.

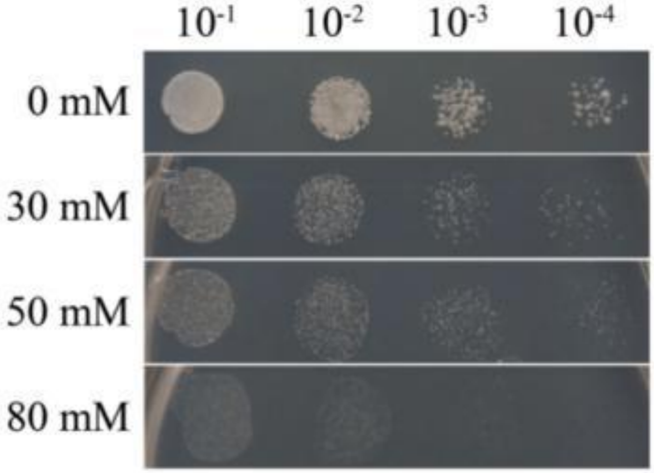

Figure S6 Screening of the optimal concentration of 3-AT for inhibiting the synthesis of background His. The yeast cells containing combination plasmid aswere serially diluted 10, 100, 1 000, and 10 000 folds, and then cultured on SD/-Trp/-Leu/-His/-Ade medium, respectively.

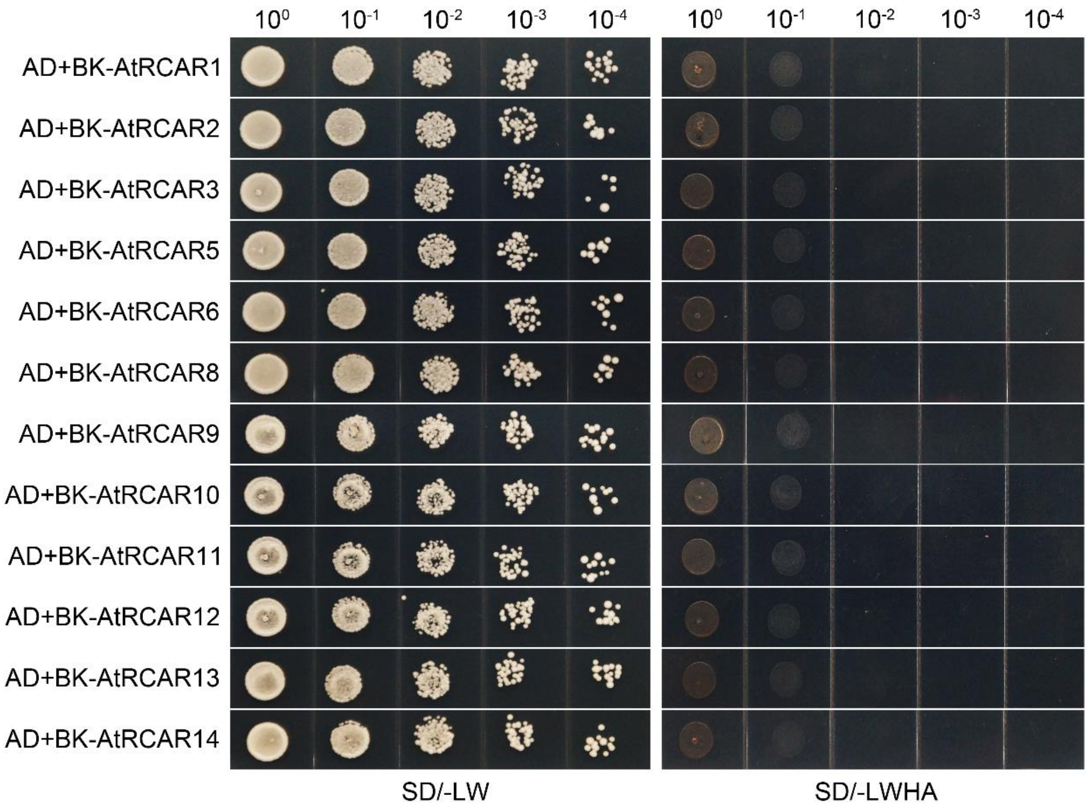

Figure S7 Validation of self-activation of AtRCARs protein. The yeast cells containing combination plasmid aswere serially diluted 10, 100, 1 000, and 10 000 folds, and then cultured on SD/-Trp/-Leu (SD/-LW) medium and SD/-Trp/-Leu/-His/-Ade (SD/-LWHA) medium, respectively.

Supplementary Tables

Table S1 Genes primers used in this study

Primer name	Primer Sequence 5’ to 3’
FtMYB22-F	ATGAAGCCATTTCCAAACAAG
FtMYB22-F	CCATAGACCACCCAGGGTC
FtMYB22-GFP-F	tctccccttgctccgtATGAAGCCATTTCCAAACAAGG
FtMYB22-GFP-R	tacccccgggtctagaCCATAGACCACCCAGGGTCC
pBridge-FtMYB22-F	acagttgactgtatcgccggaaATGAAGCCATTTCCAAACAAGG
pBridge-FtMYB22-R	tcgcccggaattagcttggcCTACCATAGACCACCCAGGGTCC
pCAMBIA 1305-FtMYB22-F	ttttctgattaacagggtaccATGAAGCCATTTCCAAACAAGG
pCAMBIA 1305-FtMYB22-R	tttgtagtcctgcaggtcgacCCATAGACCACCCAGGGTCC
ProFtMYB22-F	AGGCCAATCTAACCGCTTTC
ProFtMYB22-R	AATTGATGTTAATGAAGTTCCA
pBI101-*ProFtMYB22*-F	tgaccatgattacgccAGGCCAATCTAACCGCTTTC
pBI101-*ProFtMYB22*-R	ccggggatcctctagaAATTGATGTTAATGAAGTTCCAGTG
pBI101-*ProFtMYB22*-1-F	cttgcatgcctgcaggtcgacaAATTCTAGAATTTTTTTTTCAGTTAA
pBI101-*ProFtMYB22*-1-R	ggactgaccacccggggaTCCAATTGATGTTAATGAAGTTCCAGT

pBI101-*ProFtMYB22*-2-F	cttgcatgcctgcaggtcgacaTTTTGTTATTGTTTATCTATTCTCTCT
pBI101-*ProFtMYB22*-2-R	ggactgaccacccggggatccAATTGATGTTAATGAAGTTCCAGT
pYES2-FtMYB22-F	gacgatgacgataaggtaccATGAAGCCATTTCCAAACAAGG
pYES2-FtMYB22-R	gctggatatctgcagaattcCTACCATAGACCACCCAGGGTC
pHis2.1-NACRS-F	ctcactatagggcgaattcTTTCGTATTGCGTGTTTCGTA
pHis2.1-NACRS-R	gattcgcgaacgcgtgagctcTACGAAACACGCAATACGAAA
pHis2.1-mNACRS-F	ctcactatagggcgaattcTTTCGCCTTGCGCGTTTCGCC
pHis2.1-mNACRS-R	gattcgcgaacgcgtgagctcGGCGAAACGCGCAAGGCGAAA
pGADT7-FtMYB22-F	taccagattacgctcatATGATGAAGCCATTTCCAAACAAG
pGADT7-FtMYB22-R	tgcccacccgggtggaattcCTACCATAGACCACCCAGGGT
pGBKT7-AtRCAR1-F	tggccatggaggccgaattcATGATGGACGGCGTTGAAGG
pGBKT7-AtRCAR1-R	tgcggccgctgcaggtcgacCTGAGTAATGTCCTGAGAAGCCAAT
pGBKT7-AtRCAR2-F	tggccatggaggccgaattcATGGAGATGATCGGAGGAGACG
pGBKT7-AtRCAR2-R	tgcggccgctgcaggtcgacAAGGTTGGTTTCTGTATGATTCT

pGBKT7-AtRCAR3-F	tggccatggaggccgaattcATGGAAGCTAACGGG ATTGAGAAC
pGBKT7-AtRCAR3-R	tgcggccgctgcaggtcgacGACTCTCGATTCTGT CGTGTCTTGA
pGBKT7-AtRCAR5-F	tggccatggaggccgaattcATGGAAACTTCTCAA AAATATCA
pGBKT7-AtRCAR5-R	tgcggccgctgcaggtcgacCAACTTTAGATGAGC CACCCTCT
pGBKT7-AtRCAR6-F	tggccatggaggccgaattcATGAAAACATCTCAA GAACAGC
pGBKT7-AtRCAR6-R	tgcggccgctgcaggtcgacAGTGAGCTCCATCAT CTTCTCC
pGBKT7-AtRCAR8-F	tggccatggaggccgaattcATGAGGTCACCGGTG CAACT
pGBKT7-AtRCAR8-R	tgcggccgctgcaggtcgacTTGCCGGTTGGTACT TCGA
pGBKT7-AtRCAR9-F	tggccatggaggccgaattcATGCCAACGTCGATA CAGTTTC
pGBKT7-AtRCAR9-R	tgcggccgctgcaggtcgacCGAGAATTTAGAAGT GTTCTCGG
pGBKT7-AtRCAR10-F	tggccatggaggccgaattcATGCTTGCCGTTCAC CGTC
pGBKT7-AtRCAR10-R	tgcggccgctgcaggtcgacCAGAGACATCTTCTT CTTGCTCTCA
pGBKT7-AtRCAR11-F	tggccatggaggccgaattcATGCCTTCGGAGTTA ACACCA
pGBKT7-AtRCAR11-R	tgcggccgctgcaggtcgacCGTCACCTGAGAACC ACTTCC

pGBKT7-AtRCAR12-F	tggccatggaggccgaattcATGGCGAATTCAGAG TCCTCC
pGBKT7-AtRCAR12-R	tgcggccgctgcaggtcgacCCTAACCTGAGAAG AGTTGTTGTTG
pGBKT7-AtRCAR13-F	tggccatggaggccgaattcATGAATCTTGCTCCA ATCCATG
pGBKT7-AtRCAR13-R	tgcggccgctgcaggtcgacGGTCGGAGAAGCCG TGGAAA
pGBKT7-AtRCAR14-F	tggccatggaggccgaattcATGAGCTCATCCCCG GCCGTG
pGBKT7-AtRCAR14-R	tgcggccgctgcaggtcgacTTCATCATCATGCAT AGGTGC
qActin2-F	CTGGAATGGTGAAGGCTGGTT
qActin2-R	CGATTGGATACTTCAGAGTGAGGAT
qFtH3-F	GAAATTCGCAAGTACCAGAAGAG
qFtH3-R	CCAACAAGGTATGCCTCAGC
qFtMYB22-F	ACAACATTTACCAGGGAGGACAGAT
qFtMYB22-R	AAAGAAGACCCAGATTCCTGATAAC
qAtRD29A-F	GTCTGCCGTGACGACGAAGTTAC
qAtRD29A-R	TCCTTCTTCTCTTCTTCTCCTCCAA
qAtRD29B-F	GCAAGCAGAAGAACCAATCA
qAtRD29B-R	CTTTGGATGCTCCCTTCTCA
qAtRD22-F	GGTTCGGAAGAAGCGGAG
qAtRD22-R	GAAACAGCCCTGACGTGATAT

qAtDREB2A-F	GGTAAAGGAGGACCAGAGAATAGCC
qAtDREB2A-R	AGACGAGCCAAAGGACCATACATAG
qAtP5CS2-F	ATGGTGGCGTTCCTGTTATC
qAtP5CS2-R	GGCATTGGCAATGTCGTG
qAtPP2CA-F	TCTGAGAATCATCATTTCTACGGTGT
qAtPP2CA-R	CTCGTTGGCTAACTTCTTTATCCATT
qAtKIN1-F	GGACCAACAAGAATGCCTTCCAAGC
qAtKIN1-R	CGCTGCCGCATCCGATACACT
qAtCOR15A-F	GGTAAAGCAGGAGAGGCTAAGGATG
qAtCOR15A-R	AAGAATGTGACGGTGACTGTGGATA

Table S2 Predicted cis-elements of the *ProFtMYB22*

Element	function	number
G-Box	ethylene-responsive element	6
HSE	cis-acting element involved in heat stress responsiveness	6
Box I	light responsive element	5
Circadian	cis-acting regulatory element involved in circadian control	4
as-2-box	involved in shoot-specific expression and light responsiveness	3
ACE	cis-acting element involved in light responsiveness	2
ARE	cis-acting regulatory element essential for the anaerobic induction	2
Box-W1	fungal elicitor responsive element	2
CGTCA-motif	cis-acting regulatory element involved in the MeJA-responsiveness	2
ERE	elicitor-responsive element	2
GA-motif	part of a light responsive element	2
I-box	part of a light responsive element	2
Skn-1_motif	cis-acting regulatory element required for endosperm expression	2
Sp1	light responsive element	2
TC-rich repeats	cis-acting element involved in defense and stress responsiveness	2
TCT-motif	part of a light responsive element	2
ABRE	cis-acting element involved in the abscisic acid responsiveness	1
AE-box	part of a module for light response	1

AuxRR-core	cis-acting regulatory element involved in auxin responsiveness	1
Box III	protein binding site	1
CCAAT-box	MYBHv1 binding site	1
GCN4_motif	cis-regulatory element involved in endosperm expression	1
H-box	cis-acting regulatory element involved in light responsiveness	1
LAMP-element	part of a light responsive element	1
MBS	MYB binding site involved in drought-inducibility	1
MRE	part of a light responsive element	1
TCCC-motif	part of a light responsive element	1
TGA-element	auxin-responsive element	1
Circadian	cis-acting regulatory element involved in circadian control	4
